# SESN1 functions as a new tumor suppressor gene via Toll‐like receptor signaling pathway in neuroblastoma

**DOI:** 10.1111/cns.14664

**Published:** 2024-03-22

**Authors:** Zhongyan Hua, Bo Chen, Baocheng Gong, Meizhen Lin, Yifan Ma, Zhijie Li

**Affiliations:** ^1^ Department of Pediatrics Shengjing Hospital of China Medical University Shenyang China; ^2^ Liaoning Key Laboratory of Research and Application of Animal Models for Environmental and Metabolic Diseases, Medical Research Center Shengjing Hospital of China Medical University Shenyang China

**Keywords:** MYCN, neuroblastoma, SESN1, TLR signaling pathway, tumor suppressor gene

## Abstract

**Aims:**

Neuroblastoma (NB) is the most common extracranial solid tumor in children, with a 5‐year survival rate of <50% in high‐risk patients. MYCN amplification is an important factor that influences the survival rate of high‐risk patients. Our results indicated MYCN regulates the expression of SESN1. Therefore, this study aimed to investigate the role and mechanisms of SESN1 in NB.

**Methods:**

siRNAs or overexpression plasmids were used to change MYCN, SESN1, or MyD88's expression. The role of SESN1 in NB cell proliferation, migration, and invasion was elucidated. Xenograft mice models were built to evaluate SESN1's effect in vivo. The correlation between SESN1 expression and clinicopathological data of patients with NB was analyzed. RNA‐Seq was done to explore SESN1's downstream targets.

**Results:**

SESN1 was regulated by MYCN in NB cells. Knockdown SESN1 promoted NB cell proliferation, cell migration, and cell invasion, and overexpressing SESN1 had opposite functions. Knockdown SESN1 promoted tumor growth and shortened tumor‐bearing mice survival time. Low expression of SESN1 had a positive correlation with poor prognosis in patients with NB. RNA‐Seq showed that Toll‐like receptor (TLR) signaling pathway, and PD‐L1 expression and PD‐1 checkpoint pathway in cancer were potential downstream targets of SESN1. Knockdown MyD88 or TLRs inhibitor HCQ reversed the effect of knockdown SESN1 in NB cells. High expression of SESN1 was significantly associated with a higher immune score and indicated an active immune microenvironment for patients with NB.

**Conclusions:**

SESN1 functions as a new tumor suppressor gene via TLR signaling pathway in NB.

## Graphical abstract

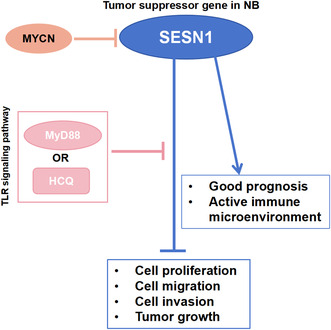

## 1 INTRODUCTION

Neuroblastoma (NB), the most common extracranial solid tumor in children, originates from sympathetic neuronal crest cells and exhibits a clinical and biological heterogeneity character.^1^, ^2^, ^3^ Patients with NB are assigned to different risk groups based on the biologic and genetic characteristics and classic clinical staging. Patients at low and intermediate risk levels have a 5‐year survival rate of approximately 90%, whereas high‐risk patients have a 5‐year survival rate of <50%.^1^, ^4^ There are about 20%–30% of high‐risk patients have MYCN amplification, and approximately half of high‐risk patients have metastasis, with the bone marrow, bone, and liver being the most common sites of metastasis.^1^, ^4^, ^5^, ^6^, ^7^ High‐risk patients with MYCN amplification or metastasis have an extremely poor prognosis. Therefore, it is crucial to elucidate the potential molecular mechanisms of how MYCN function in NB and NB metastasis to improve the survival rate of patients.

SESN1 (also known as PA26), a P53 target gene, together with SESN2 and SESN3 are the Sestrin family members.^8^, ^9^ To date, the functions of SESNs are not well understood. According to the present studies, Sestrin family members can be activated via various stresses, including oxidative stress and DNA damage, and SESN1 can be activated in a p53‐dependent manner. Additionally, SESN1, SESN2, and SESN3 can be activated by the transcription factor forkhead transcription factor (FoxO3A) and FoxO1.^10^, ^11^, ^12^, ^13^, ^14^ Moreover, SESN2 and SESN3 have been reported involved in anoikis resistance of endometrial cancer cell lines, which are regulated by miRNAs.^15^ In this study, we explored the function of SESN1 in NB.

Toll‐like receptors (TLRs) are a family of receptors that signaling via MyD88‐dependent or MyD88‐independent axes to target on its downstream targets.^16^, ^17^ TLRs are divided into two categories: cell surface TLRs (TLR1, TLR2, TLR4, TLR5, TLR6, and TLR10) and intracellular TLRs (TLR3, TLR7, TLR8, TLR9, TLR11, TLR12, and TLR13).^17^, ^18^, ^19^ Although these TLRs function via MyD88‐dependent and MyD88‐independent axes, MyD88 is the common adaptor for all TLRs.^18^ It has been known that TLR signaling pathways play important roles in innate immunity,^18^, ^19^ recent studies also showed that TLRs regulate cell survival, epithelial‐mesenchymal transition (EMT), and tumor cell invasion.^17^, ^20^, ^21^ Furthermore, KEGG enrichment analysis base on our RNA‐seq results showed that TLR signaling pathway was one of the pathways that were enriched in all of the four NB cell lines after knockdown the expression of SESN1. Based on the above, in this study, we investigated whether SESN1 plays a role in NB through the MyD88‐dependent TLR signaling pathway.

In this study, we demonstrate that MYCN regulated the expression of SESN1 in NB cells, and SESN1 regulated the cell proliferation, cell migration, and invasion of NB cells in vitro, and tumor growth and NB tumor‐bearing mice survival in vivo. Additionally, we found that low expression of SESN1 means poor prognosis for NB patients, and the function of SESN1 in NB was mediated by MyD88‐dependent TLR signaling pathway. Our study indicates SESN1 functions as a new tumor suppressor gene via a MyD88‐dependent TLR signaling pathway in NB, and SENS1 and TLR signaling pathway may be new therapy targets for NB clinical treatment. And, we also found that high expression of SESN1 indicated an active immune microenvironment, which indicated SESN1 could be a potential immunotherapeutic target.

## 2 MATERIALS AND METHODS

### 2.1 Cell lines and cell culture

Four NB cell lines (SK‐N‐AS (AS), NGP, SK‐N‐BE2 (BE2), and SH‐SY5Y (SY5Y)) were used in this study. AS and SY5Y cell lines are non‐MYCN‐amplified, NGP and BE2 are MYCN‐amplified. All the four cell lines are a gift from Dr. Carol J. Thiele (Cellular and Molecular Biology Section, Pediatric Oncology Branch, National Cancer Institute, National Institutes of Health, Bethesda, MD, USA). NB cells were cultured in RPMI‐1640 medium (Hyclone, USA) containing 10% fetal bovine serum (Gibco, USA), 100 U/mL penicillin, 100 mg/mL streptomycin (Biological Industries, Israel) and 2 mM/L glutamine (Biological Industries, Israel) at 37°C in a 5% CO_2_ incubator.

### 2.2 Reagents and antibodies

Primary antibodies anti‐MYCN and anti‐SESN1 were purchased from Abcam (UK), and anti‐MyD88, anti‐GAPDH, and anti‐β‐Tubulin were purchased from Proteintech (Wuhan, Hubei, China). Cell Counting Kit‐8 (CCK‐8) was bought from Bimake (Shanghai, China). Matrigel was bought from Corning (NY, USA). HiSpeed Plasmid Maxi Kit was bought from Qiagen (Germany). jetPRIME was purchased from PolyPlus Transfection Co. (Illkirsch, France). TLR signaling pathway inhibitor Hydroxychloroquine‐d4 sulfate (HCQ) was purchased from MCE (USA).

### 2.3 Identifying hub genes after MYCN‐knockout in NB cell

The CRISPR‐Cas9 knockout MYCN microarray dataset in NB cell was obtained from gene expression omnibus (GEO) (http://www.ncbi.nlm.nih.gov/geo/) at GSE121529.^22^ Protein–protein interaction (PPI) network was generated to identify the interacting genes using STRING (https://string‐db.org), and Cytoscape software was used to calculate the node degree using the Network Analyzer application. Gene ontology (GO) enrichment analysis and Kyoto Encyclopedia of Genes and Genomes (KEGG) pathway analysis of differentially expressed genes (DEGS) were performed using the DAVID (http://david.abcc.ncifcrf.gov/) website. Statistical significance was set at *p* < 0.05.

### 2.4 Cell transfection

Small interfering RNAs (siRNAs) were purchased from Tongyong (Anhui, China) and used to knock down SESN1 or MyD88. The sequences of siRNAs were as follows:

SESN1‐siRNA#1 sense: GGAUCAAGGCGGAGAGGAATT;

SESN1‐siRNA#1 anti‐sense: UUCCUCUCCGCCUUGAUCCTT;

SESN1‐siRNA#2 sense: CCAAGAACGUGACAGAUGATT;

SESN1‐siRNA#2 anti‐sense: UCAUCUGUCACGUUCUUGGTT.

MyD88‐siRNA#1 sense: CGUUGUAGGAGGAAUCUGUTT;

MyD88‐siRNA#1 anti‐sense: ACAGAUUCCUCCUACAACGTT;

MyD88‐siRNA#2 sense: CUCUACUUACCUCUCAAUUTT;

MyD88‐siRNA#2 anti‐sense: AAUUGAGAGGUAAGUAGAGTT.

SESN1 expression plasmid purchased from GenePharma (Shanghai, China) was used to overexpression SESN1. The information of MYCN siRNAs and MYCN expression plasmid is the same as previously described.^23^ NB cells were seeded into 6‐well plate (AS: 2 × 10^5^/well, BE2, NGP, and SY5Y: 5 × 10^5^/well). After cultured overnight, siRNAs or overexpression plasmids were transfected into cells using jetPRIME.

### 2.5 Cell viability assay

NB cells (AS, BE2, NGP, and SY5Y) were seeded into 96‐well plate 16 h after transfection with siRNAs or overexpression plasmids. The plate was incubated in the IncuCyte ZOOM (Essen BioSciences) equipment, which was scanned every 4 h, and the percentage of cell confluence was measured and analyzed by IncuCyte ZOOM software based on the images which was used to indicate cell survival. The points showed three replicates and presented as means ± standard deviation (SD). At the end of the experiment, CCK‐8 assay was performed to detect the cell survival according to the manufacturer's introduction.

### 2.6 Colony formation assay

Single‐cell suspensions were seeded into 6‐cm dishes 16 h after transfection with siRNAs or overexpression plasmids. When the single‐cell colonies were visible to the naked eye, the cells were fixed with methyl alcohol, and stained with 1 × Giemsa working solution (diluted from 10× stock solution with PBS) for 30 min at room temperature. After gently washing with water, dishes with dyed colonies were dried, and images were captured under a microscope. Colonies were counted using Image J software as described previously.^24^

### 2.7 In vivo studies

NB cells (AS, BE2, NGP, and SY5Y) were harvested 16 h after being transfected with SESN1 siRNAs, and washed with PBS, and resuspended in PBS and Matrigel (1:1, v/v). AS (1.5 × 10^6^ cells), NGP, BE2, or SY5Y (2 × 10^6^ cells) cells in 100 μL suspensions were inoculated into the subcutaneous tissue of the right flank of 4‐5 week‐old female nude mice (Beijing Huafukang Bioscience Co. Inc., China) (AS: 7 mice/group; SY5Y: 11 mice/group; BE2: 10–13 mice/group; NGP: 12 mice/group). The weights of mice were measured once per week. The tumor volume (V) was measured three times a week using a digital caliper from the appearance of tumor to the endpoint of the experiment and was calculated as L × W^2^/2 (L = length, millimeter; W = width, millimeter). To detect the effect of SESN1 on the survival of tumor‐bearing mice, we counted the days from the date of tumor cell injection to the endpoint of the experiment (including natural mortality and euthanasia according to the ethical principles). At the end of the experiment, the tumors were excised and immediately frozen at −80°C. All experiments involving animals were approved by the Experimental Animal Ethics Committee of Shengjing Hospital of China Medical University (2021PS558K).

### 2.8 Western blotting

To evaluate the expression of MYCN, SESN1, and MyD88 in NB cells or NB tumor tissues, protein lysates were extracted by using RIPA buffer (Beyotime, China) with protease inhibitors cocktail and PhosphoSafe (Bimake, China). Thereafter, 30 μg protein from NB cells or 50 μg protein from NB tumor tissues was loaded, and then western blotting was carried out as described previously.^25^

### 2.9 Scratch wound healing assay

NB cells (AS, BE2, NGP, and SY5Y) were seeded into 96‐well plate 16 h after transfection with siRNAs or overexpression plasmids. After 24 h, the cell monolayer was scratched with a 200‐μl pipette tip across the center of the well, followed by incubation in the IncuCyteZOOM equipment. Images were captured every 4 h, and the relative wound density was analyzed to calculate the cell migration rate, using the IncuCyteZOOM software.

### 2.10 Boyden chamber migration and invasion assays

NB cells (AS, BE2, NGP, and SY5Y) were harvested in 5% FBS RPMI‐1640 media for 16 h after transfection with siRNAs or overexpression plasmids. Thereafter, 100‐μL of NB cell suspension (10 × 10^4^ cells/insert) was seeded in a 24‐well Boyden chamber trans‐well insert (Corning, NY, USA) for the migration or invasion assay. The Boyden chamber trans‐well insert has an 8.0‐μm‐pore polyethylene terephthalate membrane at the bottom, which was pre‐coated with 30 μL of Matrigel (1:10 diluted with plain media) for invasion assay or left uncoated for migration assay. Bottom wells were fed with 600 μL of 15% FBS RPMI‐1640 media. After 48 h, non‐migrating or non‐invading cells were removed by scraping the upper side of the insert using a cotton‐tipped applicator as described previously.^26^ The cells that migrated or invaded to the underside of the insert were stained with 1 × Giemsa working solution at room temperature. Photographs of three random fields were taken, and cells were counted to calculate the average number of migratory or invasive cells per well.

### 2.11 RNA sequencing and bioinformatic analysis

NB cells (AS, BE2, NGP, and SY5Y) were transfected with Control‐siRNA or SESN1 siRNAs (SESN1‐siRNA#1 and #2) and were harvested 16 h after transfection. Total RNA for RNA sequencing was extracted using TRiZol (Sigma, USA). RNA sequencing was performed by Gene Denovo Co. (Guangzhou, China). Differential expression analysis was performed to identify DEGs using R language. DEGs were identified at a cut‐off of *p* value <0.05, FDR <0.05, and |log_2_FC| > 2, and heatmaps of DEGs were generated using the R language. KEGG pathway analysis of DEGS was performed using DAVID (http://david.abcc.ncifcrf.gov/).

### 2.12 Clinical datasets source and data analysis

Clinical datasets (NBL datasets, E‐MTAB‐8284 datasets, and GSE40710 datasets) were downloaded from TARGET (https://www.cancer.gov/ccg/research/genome‐sequencing/target), ArrayExpress (https://www.ebi.ac.uk/biostudies/arrayexpress), and GEO (https://www.ncbi.nlm.nih.gov/geo/) databases, and the gene expression profile, clinicopathological data, and prognostic information of patients with NB in each dataset were collated.

The correlation between the expression of SESN1 and the overall survival of patients with NB was analyzed using the “survival” and “survminer” packages in R language (Kaplan–Meier survival analyses). The correlation between the expression of SESN1 and clinicopathological data of patients with NB was analyzed using the “limma” and “ComplexHeatmap” packages in R, and the correlation heatmap was plotted. Data analysis was performed using a one‐way ANOVA.

The “CIBERSORT” algorithm (http://cibersort.stanford.edu/), which is based on 22 human immune cell phenotypes (B cells, T cells, NK cells, macrophages, dendritic cells (DCs), and myeloid subsets), was used to identify the proportions of immune cells in NB tumor samples. According to the median value of SESN1 gene expression, the NB tumor samples were divided into two groups, and the immune cell contents in the two groups were compared using the Wilcoxon signed‐rank test. Tumor immune dysfunction and exclusion (TIDE; http://tide.dfci.harvard.edu/) algorithms were used to explore the efficacy of immunotherapy.

### 2.13 Statistical analysis

Data were expressed as mean ± standard deviation (SD). Shapiro–Wilk test for normality was used to assess data distribution. Statistical comparisons between the two groups were carried out with two‐tailed Student's *t*‐test. The Kaplan–Meier method was used to determine the survival of mice. Statistical analysis was performed using the GraphPad Prism software. *p* Values <0.05 were considered statistically significant.

## 3 RESULTS

### 3.1 Knockdown the expression of SESN1 increased NB cell proliferation

**FIGURE 1:**
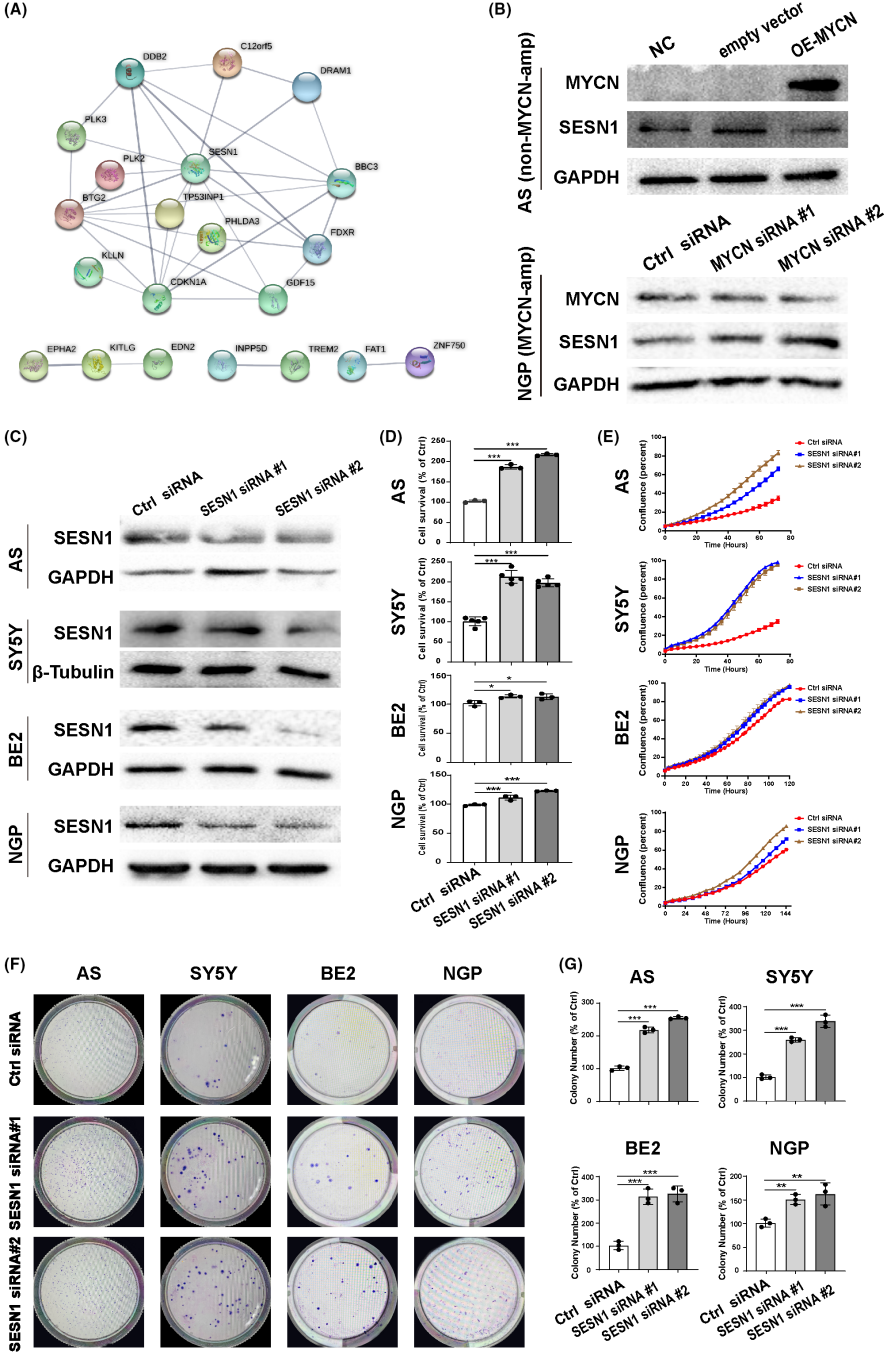
Knockdown SESN1 promote cell proliferation of NB cells. (A) PPI network analysis of the DEGs in MYCN‐knockout NB cells. (B) Western blotting was performed to detect the expression of MYCN and SESN1 in MYCN siRNAs‐transfected BE2 cells (MYCN‐amplified) and MYCN expression plasmid‐transfected AS cells (non‐MYCN‐amplified). (C) The expression of SESN1 was detected in SESN1 siRNAs‐transfected NB cells (AS, SY5Y, BE2, NGP). (D) AS, SY5Y, BE2, and NGP cells were seeded into 96‐well plate 16 h after being transfected with SESN1 siRNAs, followed by a CCK‐8 assay to detect cell survival. The percentage of surviving cells was calculated by normalizing with control siRNA‐transfected cells. *p < 0.05, ***p < 0.001, control siRNA‐transfected cells versus SESN1 siRNA#1 or #2‐transfected cells. (E) SESN1 siRNAs‐transfected NB cells in 96‐well plates were cultured in IncuCyte ZOOM, and the cell confluence was calculated based on the phase‐contrast images. SESN1 siRNAs‐transfected NB cells were seeded in 6 cm dishes for colony formation assay. Respective images are shown (F), and statistical analysis was performed (G), **p < 0.01, ***p < 0.001, control siRNA‐transfected cells versus SESN1 siRNA#1 or #2‐transfected cells.

MYCN is an oncogene, whose amplification means poor prognosis in NB. Hiroyuki Yoda et al. developed a CRISPR‐Cas9 system to knockout MYCN expression in NB cells and then performed a microarray (GSE121529).^22^ Based on the microarray results, we performed a PPI network analysis of the DEGS using STRING (https://cn.string‐db.org/) and calculated the node degree using Cytoscape software. Results showed that SESN1 may be a core regulatory gene involved in the function of MYCN in NB (Figure 1A and Figure S1A). Furthermore, knockdown the expression of MYCN in NGP cells (MYCN‐amplified) increased the expression of SESN1, and overexpression of MYCN in AS cells (non‐MYCN‐amplified) decreased the expression of SESN1 (Figure 1B). GO functional annotation and KEGG enrichment analysis showed that SESN1 plays important roles in cell division, cell proliferation, cell cycle, and p53 signaling pathway (Figure S1B). Furthermore, we analyzed the relationship between SESN1 and the survival probability of patients with NB using the R2, NBL, E‐MTAB‐8284, and GSE40710 datasets and found that patients with high expression of SENS1 had better Event‐free and overall survival probability than patients with low expression of SESN1 (Figure S1C,D).

To explore the role of SESN1 in NB, we developed two siRNAs to downregulate the expression of SESN1. The expression of SESN1 decreased in all of the four NB cell lines (AS, SY5Y, BE2, NGP) after transfection with siRNAs (Figure 1C). The results of CCK8 assay and cell confluence detected by IncuCyte ZOOM showed that knockdown the expression of SESN1 significantly increased NB cell proliferation, especially in AS and SY5Y cells (non‐MYCN‐amplified) (Figure 1D,E). Additionally, a colony formation assay was performed to confirm the effect of SESN1 on NB cell proliferation. Results showed that SESN1 downregulated group had more and bigger colonies than control group in all the four NB cell lines (Figure 1F,G). Unlike the results of CCK8 assay and cell confluence analysis, the effect of downregulation of SESN1 on colony formation was similar in all the four NB cell lines.

### 3.2 Knockdown the expression of SESN1 increased NB cell migration and invasion

**FIGURE 2:**
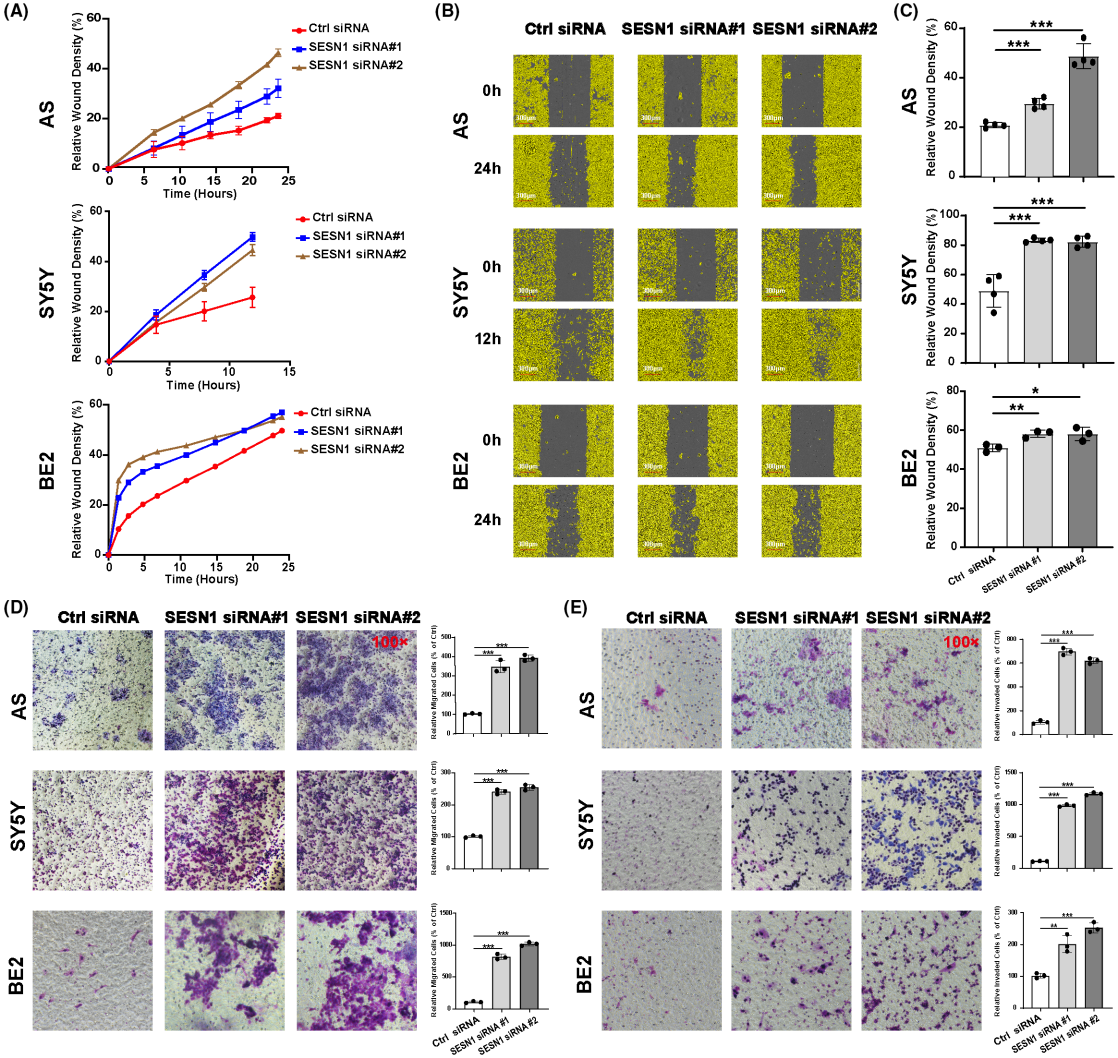
Knockdown SESN1 promote cell migration and invasion of NB cells. AS, SY5Y, and BE2 cells were seeded into 96‐well plate 16 h after transfected with SESN1 siRNAs, and cultured for 24 h, followed by wound healing assay to detect cell migration ability. Relative wound density (%) was calculated based on the phase‐contrast images using IncuCyte ZOOM software (A), respective images are shown (B), and statistical analysis was performed (C), **p < 0.01, *p < 0.05, control siRNA‐transfected cells versus SESN1 siRNA#1 or #2‐transfected cells. Trans‐well migration assay (D) and invasion assay (E) were performed, respective images are shown (left panel), and statistical analysis was performed (right panel), **p < 0.01, ***p < 0.001, control siRNA‐transfected cells versus SESN1 siRNA#1 or #2‐transfected cells.

To further detect the function of SESN1 in NB cells, we performed wound healing assay, trans‐well migration assay, and trans‐well invasion assay after downregulation of SESN1. The relative wound density of NB cells (AS, SY5Y, and BE2) transfected with SESN1 siRNAs was significantly increased compared to NB cells transfected with control siRNA (Figure 2A–C). Similar to wound healing assay, the results of trans‐well migration assay showed that the relative migration rate significantly increased after transfecting SESN1 siRNAs into NB cells (Figure 2D). And the trans‐well invasion assay showed that knockdown the expression of SESN1 increased the NB cells' (AS, SY5Y, and BE2) relative invasion rate significantly (Figure 2E). The effect of downregulated SESN1 on cell migration and cell invasion was similar in all the three NB cell lines.

### 3.3 Overexpression of SESN1 decreased cell proliferation, cell migration, and cell invasion in NB cells

**FIGURE 3:**
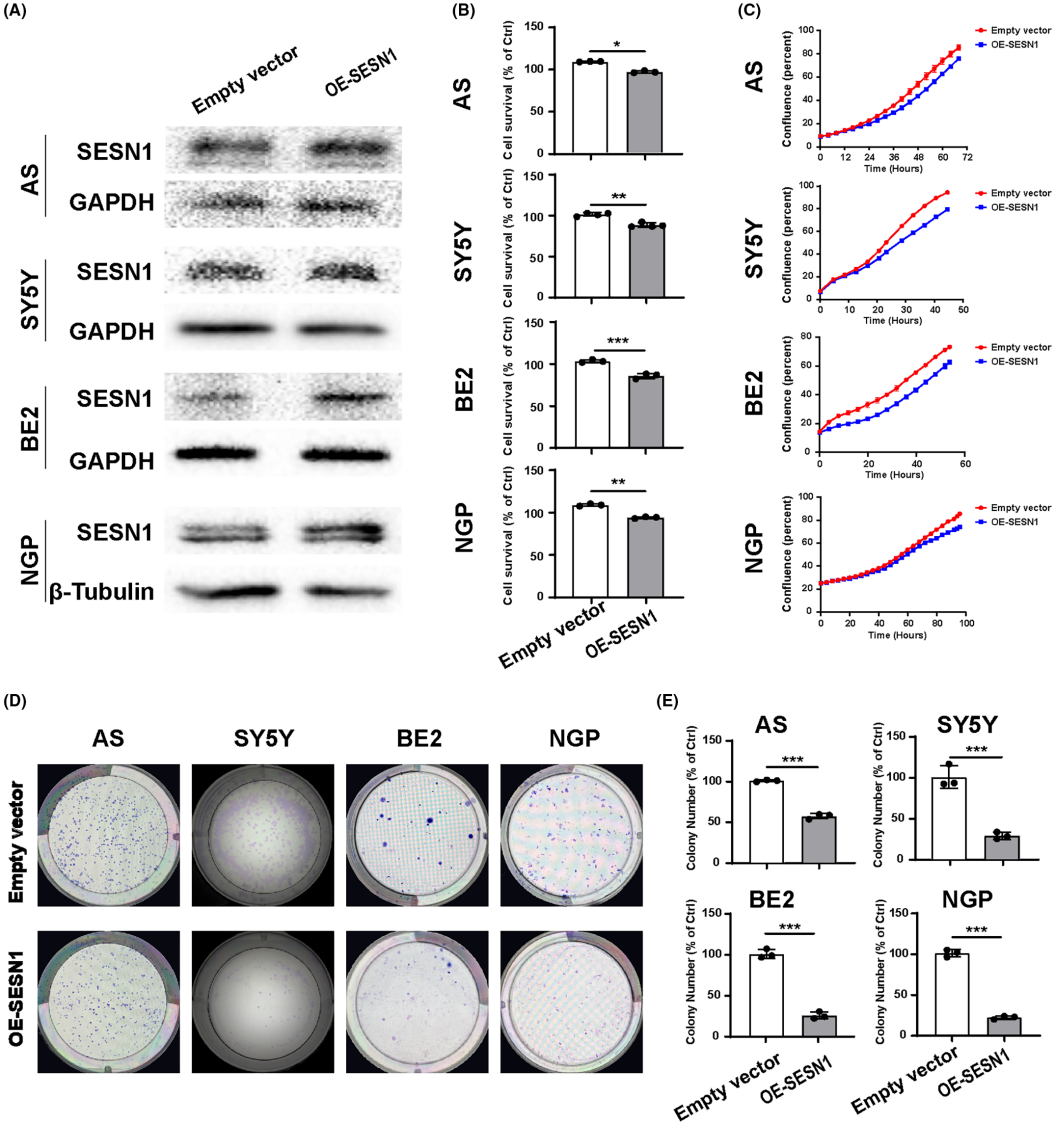
Overexpression of SESN1 inhibits cell proliferation of NB cells. (A) The expression of SESN1 was detected in NB cells (AS, SY5Y, BE2, NGP) transfected with SESN1 expression plasmid. (B) AS, SY5Y, BE2, and NGP cells were seeded into 96‐well plate 16 h after transfected with SESN1 expression plasmid, followed by CCK‐8 assay to detect cell survival, and the percentage of surviving cells was calculated by normalizing with empty vector‐transfected cells. *p < 0.05, **p < 0.01, ***p < 0.001, empty vector‐transfected cells versus SESN1 expression plasmid‐transfected cells. (C) SESN1 expression plasmid‐transfected NB cells in 96‐well plates were cultured in IncuCyte ZOOM, and the cell confluence was calculated based on the phase‐contrast images. SESN1 expression plasmid‐transfected NB cells were seeded in 6 cm dishes for colony formation assay. Respective images are shown (D), and statistical analysis was performed (E), ***p < 0.001, empty vector‐transfected cells versus SESN1 expression plasmid‐transfected cells.

**FIGURE 4:**
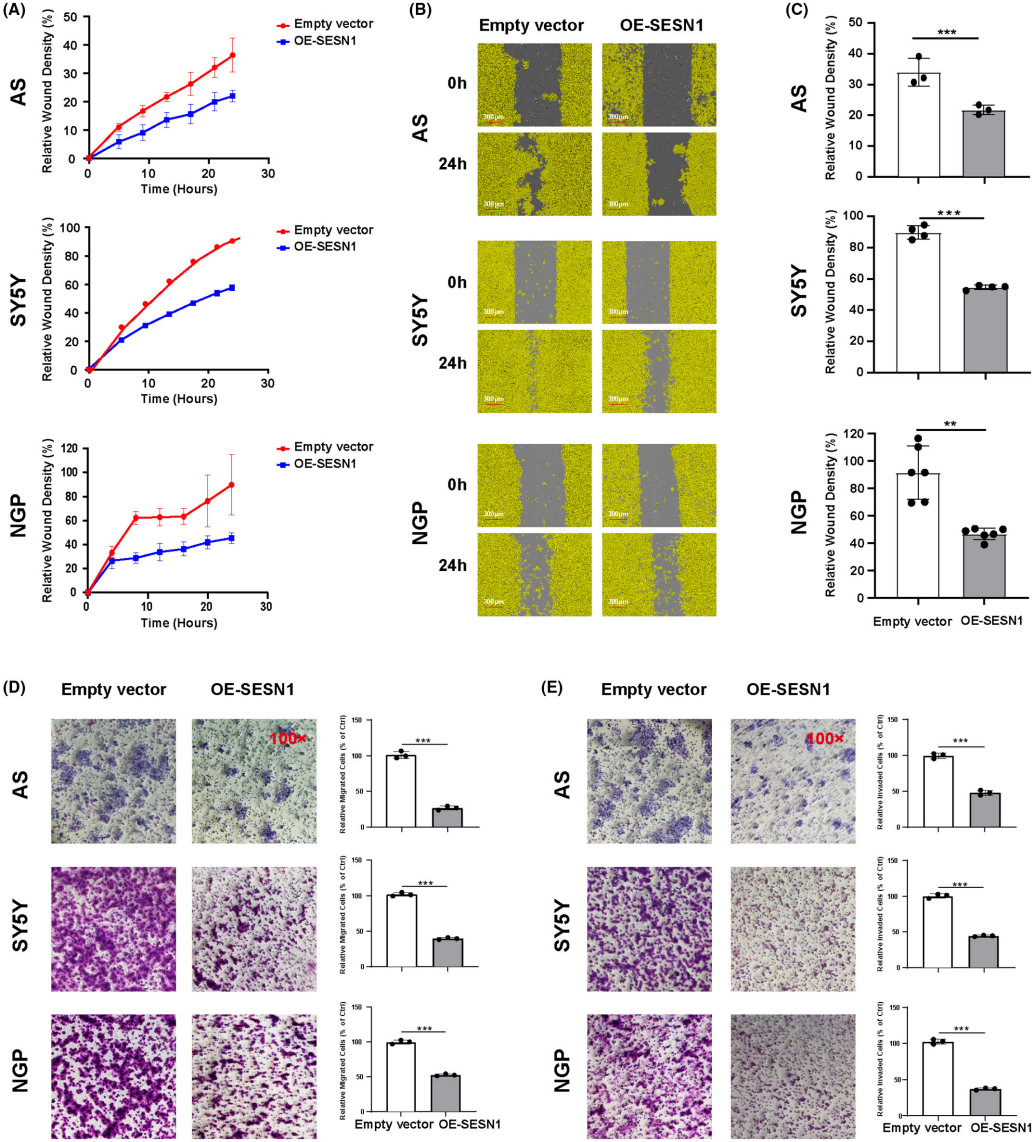
Overexpression of SESN1 inhibits cell migration and invasion of NB cells. AS, SY5Y, and NGP cells were seeded into 96‐well plate 16 h after being transfected with SESN1 expression plasmid and cultured for 24 h. This was followed by wound healing assay to detect cell migration ability. The relative wound density (%) was calculated based on the phase‐contrast images using IncuCyte ZOOM software (A), Respective images are shown (B), and statistical analysis was performed (C), ***p < 0.001, empty vector‐transfected cells versus SESN1 expression plasmid‐transfected cells. Trans‐well migration assay (D) and invasion assay (E) were performed, and respective images are shown (left panel), and statistical analysis was performed (right panel), ***p < 0.001, empty vector‐transfected cells versus SESN1 expression plasmid‐transfected cells.

To further investigate the role of SESN1 in NB, we developed SESN1 overexpression plasmids, and the expression of SESN1 can be overexpressed in all of the four NB cells (AS, SY5Y, BE2, NGP) after transfection with the plasmids (Figure 3A). The results of CCK8 assay, cell confluence detected by IncuCyte ZOOM, and colony formation assay showed that overexpression of SESN1 decreased cell proliferation of all the four NB cell lines (Figure 3B–E). Wound healing and trans‐well migration and invasion assays were performed in AS, SY5Y, and NGP cells after transfection with SESN1 overexpression plasmid. SESN1 overexpression significantly decreased the relative wound density (Figure 4A–C), relative migrated cells (Figure 4D), and relative invaded cells (Figure 4E) in all the three NB cell lines.

### 3.4 Knockdown the expression of SESN1 promoted NB tumor growth and shorted tumor‐bearing mice survival time in vivo, and low expression of SESN1 means poor prognosis for NB patients

**FIGURE 5:**
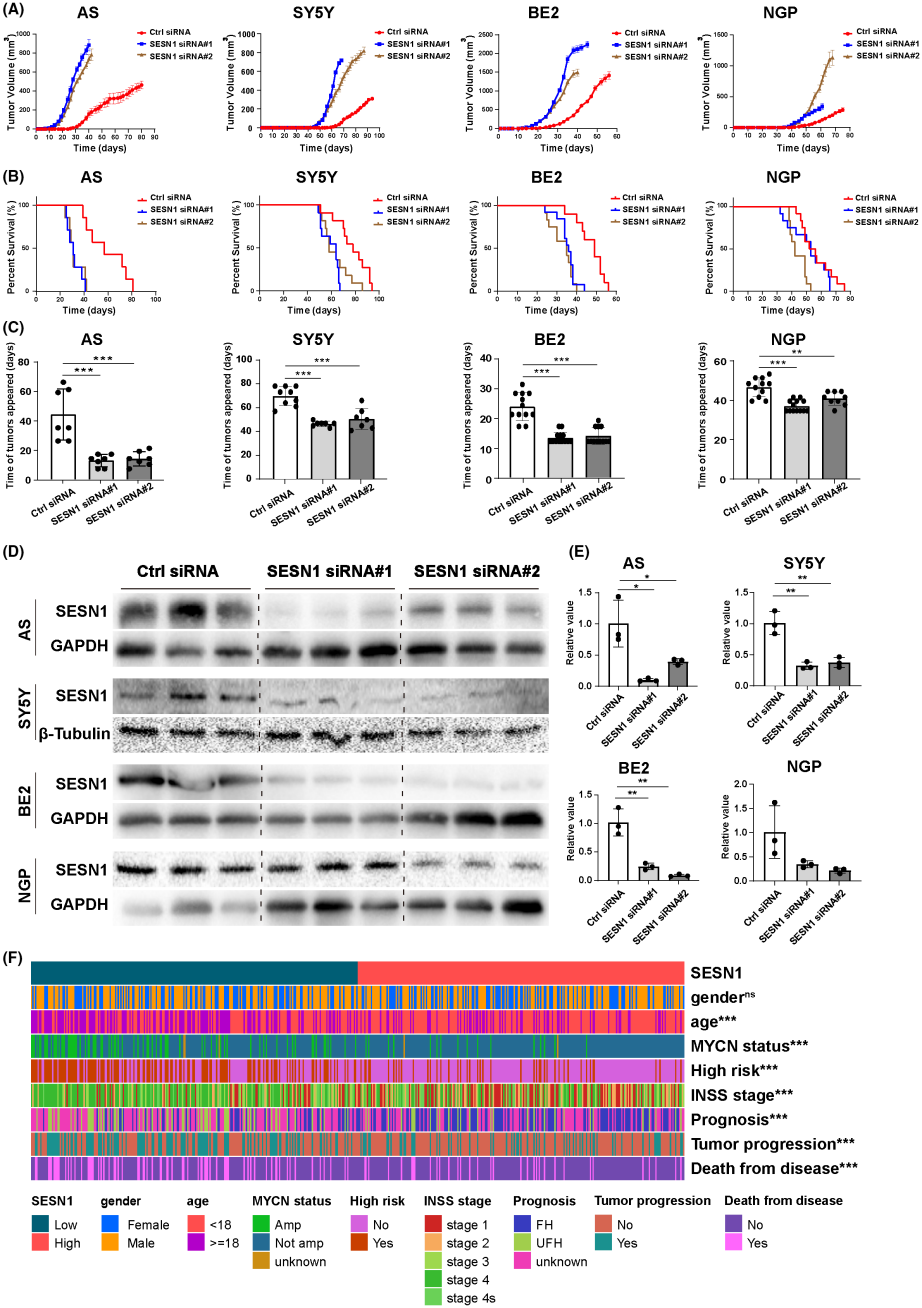
Knockdown SESN1 promotes tumor growth and decreases tumor‐bearing mice survival in vivo, and low expression of SESN1 means poor prognosis for NB patients. SESN1 siRNAs‐transfected NB cells (AS, SY5Y, BE2, and NGP) were injected into the right flank of nude mice to build xenograft mice models. (A) The mean tumor volume (mean ± standard deviation) was compared between groups and was shown according to the time of NB cell injection into mice, control siRNA‐transfected cells ‐versus SESN1 siRNA#1 or #2‐transfected cells. (B) The survival curve of tumor‐bearing mice was plotted by Kaplan–Meier analysis. (C) The mean time of tumor appeared was compared in each cell line, **p < 0.01, ***p < 0.001, control siRNA‐transfected cells versus SESN1 siRNA#1 or #2‐transfected cells. Tumor tissues were collected at the endpoint of the experiment, and the protein was extracted and the expression of SESN1 was detected using western blotting (D), and relative SESN1 value was calculated (E), *p < 0.05, **p < 0.01, control siRNA‐transfected group versus SESN1 siRNA#1 or #2‐transfected group. (F) Heatmap of the correlation between the expression levels of SESN1 and clinicopathological data of patients with NB was plotted, ns non‐significant, ***p < 0.001, patients with low expression of SESN1 versus patients with high expression of SESN1.

To evaluate the function of SESN1 in NB in vivo, we built xenograft mouse model. NB cells (AS, SY5Y, BE2, or NGP) transfected with SESN1 siRNAs or control siRNA were implanted into the right flank of BALB/c female nude mice. Compared with control siRNA‐transfected group, knockdown SESN1 promoted NB tumor growth (Figure 5A) and shortened the tumor‐bearing mice survival time (Figure 5B). Additionally, we compared the time of tumor appearance and found that tumors appeared significantly earlier in the SESN1 knockdown group than that in the control group (Figure 5C). Western blot was performed to confirm the knockdown efficiency of SESN1 siRNAs (Figure 5D,E). Furthermore, the body weight of SESN1‐knockdown mice decreased along with the tumor growth, indicating that SESN1 silencing exacerbated the disease state (Figure S2A).

Furthermore, we analyzed the correlation between the expression of SESN1 and clinicopathological data of patients with NB. The expression levels of SESN1 were not significantly different between female and male patients with NB (*p* = 0.87). While, patients with poor prognosis (age ≥18 months, MYCN‐amplification, high risk, stage3 and stage4, unfavor histopathology (UFH), with tumor progression, and death for disease) had significantly lower expression of SESN1 than those with good prognosis (*p* < 0.001) (Figure 5F and Figure S3A).

Collectively, these results indicate that SESN1 is a downstream target of MYCN and functions as a tumor suppressor gene in NB. However, the function of SESN1 has no significant difference in MYCN‐amplified and non‐MYCN‐amplified NB cells.

### 3.5 TLR signaling pathway may be the downstream target of SESN1 in NB

**FIGURE 6:**
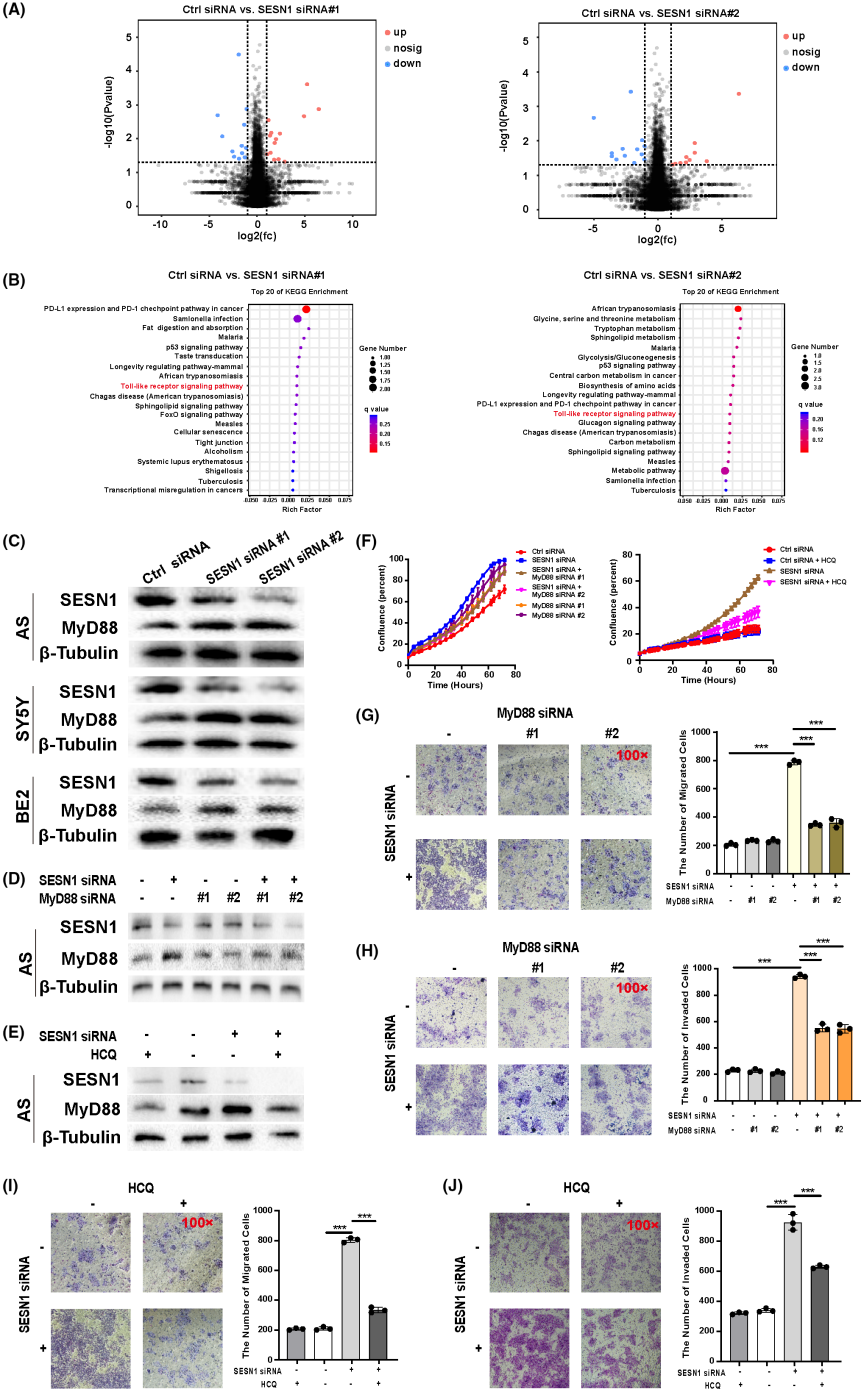
TLR signaling pathway mediates the function of SESN1 in the migration and invasion of NB cells. The RNA‐seq was performed 16 h after SESN1 siRNAs transfected into AS, SY5Y, BE2, and NGP cells. (A) The differential expressed genes (DEGs) after SESN1 siRNA#1 or siRNA#2 transfection are shown in volcano plot. (B) The KEGG pathway enrichment of DEGs was performed after SESN1 siRNA#1 or siRNA#2 transfection. (C) The expression of MyD88 in SESN1 siRNAs‐transfected NB cells (AS, SY5Y, BE2) was detected using western blotting. (D) The expressions of SESN1 and MyD88 in NB cells (AS) were detected 16 h after SESN1 siRNA#2 or/and MyD88 siRNAs transfection. (E) The expressions of SESN1 and MyD88 in NB cells (AS) were detected 16 h after SESN1 siRNA#2 transfection and/or HCQ treatment. (F) The cell confluence was calculated using IncuCyte ZOOM based on the phase‐contrast images. Trans‐well migration assay (G) and invasion assay (H) was performed after SESN1 siRNA#2 or/and MyD88 siRNAs transfection in AS cells, respective images are shown (left panel), and statistical analysis was performed (right panel), *p < 0.05, **p < 0.01, ***p < 0.001, ctrl siRNA‐transfected cells versus SESN1 siRNA#2‐transfected cells, SESN1 siRNA#2 + MyD88 siRNA#1 or #2‐transfected cells versus SESN1 siRNA#2‐transfected cells. Trans‐well migration assay (I) and invasion assay (J) were performed SESN1 siRNA#2 transfection or/and HCQ treatment in AS cells, respective images are shown, and statistical analysis was performed, **p < 0.01, ***p < 0.001, ctrl siRNA‐transfected cells versus SESN1 siRNA#2‐transfected cells, SESN1 siRNA#2 + HCQ‐treated cells versus SESN1 siRNA#2‐transfected cells.

To further explore the regulatory mechanism of SESN1 in NB, we performed RNA‐Seq of four NB cell lines (AS, SY5Y, BE2, and NGP) after transfection with SESN1 siRNAs. There are 14 upregulated (CEMP1, ADGRG1, OGN, TMEM232, FAM83H, CALB2, CALHM1, MYH7B, TXNDC2, TOGARAM2, RSPH10B, AC097637.1, S1PR4, and KCNG2) and 12 downregulated (CFAP97D1, BATF2, SPAG17, KLK14, TMC3, MTTP, CCDC154, ETV2, H3C3, RAD9B, AKAP3, and SESN1) genes with significant difference in the four NB cell lines transfection with SESN1 siRNA #1, and 10 upregulated (KCNIP3, PRRG2, AL110118.2, AHRR, TGM4, PGAM4, AC005041.1, IDO1, RSPH10B, and AC097637.1) and 13 downregulated (DNAH12, IGSF22, SGMS2, OR2B6, LIN28A, GLIPR1L2, SESN1, CCDC173, AC009070.1, CAVIN2, TBC1D3B, VWA5B2, and STXBP2) genes in the four NB cell lines transfected with SESN1 siRNA #2 (Figure 6A). KEGG enrichment analysis showed that TLR signaling pathway was one of the pathways that was enriched in all of the four cell lines transfected with SESN1 siRNA #1 and siRNA #2 (Figure 6B).

TLR signaling pathway regulates bone marrow metastasis of tumor cells.^17^, ^21^ Therefore, we hypothesized that this pathway may mediate the function of SESN1 in NB. TLR pathway activates its downstream targets via MyD88‐dependent axis or MyD88‐independent axis, indicating that MyD88 is a key factor in the activity of the TLR pathway.^17^ To confirm this hypothesis, we investigated the effect of knockdown SESN1 on MyD88 expression in NB cells (AS, SY5Y, and BE2). Knockdown SESN1 significantly upregulated MyD88 expression (Figure 6C). To further explore whether MyD88 mediated the function of SESN1 in NB cells, we developed two MyD88 siRNAs, the increased expression of MyD88 and cell confluence induced by SESN1 knockdown were blocked by MyD88 siRNAs in AS cells (Figure 6D,F). Additionally, NB cells were treated with TLR inhibitor HCQ to confirm the role of TLR pathway in NB. Cell confluence was detected after treat NB cells with different concentrations of HCQ (10, 15, 20, 25, 30 μM) (Figure S4A), and 10 μM of HCQ was used for the following experiments. Consistent with the effects of MyD88 siRNAs, HCQ treatment inhibited SESN1 knockdown‐induced increased expression of MyD88 and cell confluence in AS cells (Figure 6E,F).

Furthermore, we detected whether TLR pathway mediated the function of SESN1 on the migration and invasion of NB cells. Trans‐well migration and invasion assays were performed after transfected SESN1 siRNA or MyD88 siRNA into NB cells. Knockdown MyD88 alone had no significant effect on the number of migrated cells, but significantly blocked SESN1 knockdown‐induced increased migrated cells in all of the four NB cell lines (Figure 6G and Figure S5A). Trans‐well invasion assay was similar to the migration assay that knockdown MyD88 alone had no significant effect on the number of migrated cells, but significantly blocked SESN1 knockdown‐induced increased invaded cells in all of the four NB cell lines (Figure 6H and Figure S5B). HCQ also was used to detect whether blocking TLR signaling could reverse the function of SENS1 knockdown on the migration and invasion of NB cells. The results showed that HCQ alone had no effect on the migration and invasion of NB cells, but blocked SESN1 knockdown induced the increased migrated and invaded cells in all of the four cell lines (Figure 6I,J and Figure S5C,D).

### 3.6 High expression of SESN1 indicated an active immune microenvironment

**FIGURE 7:**
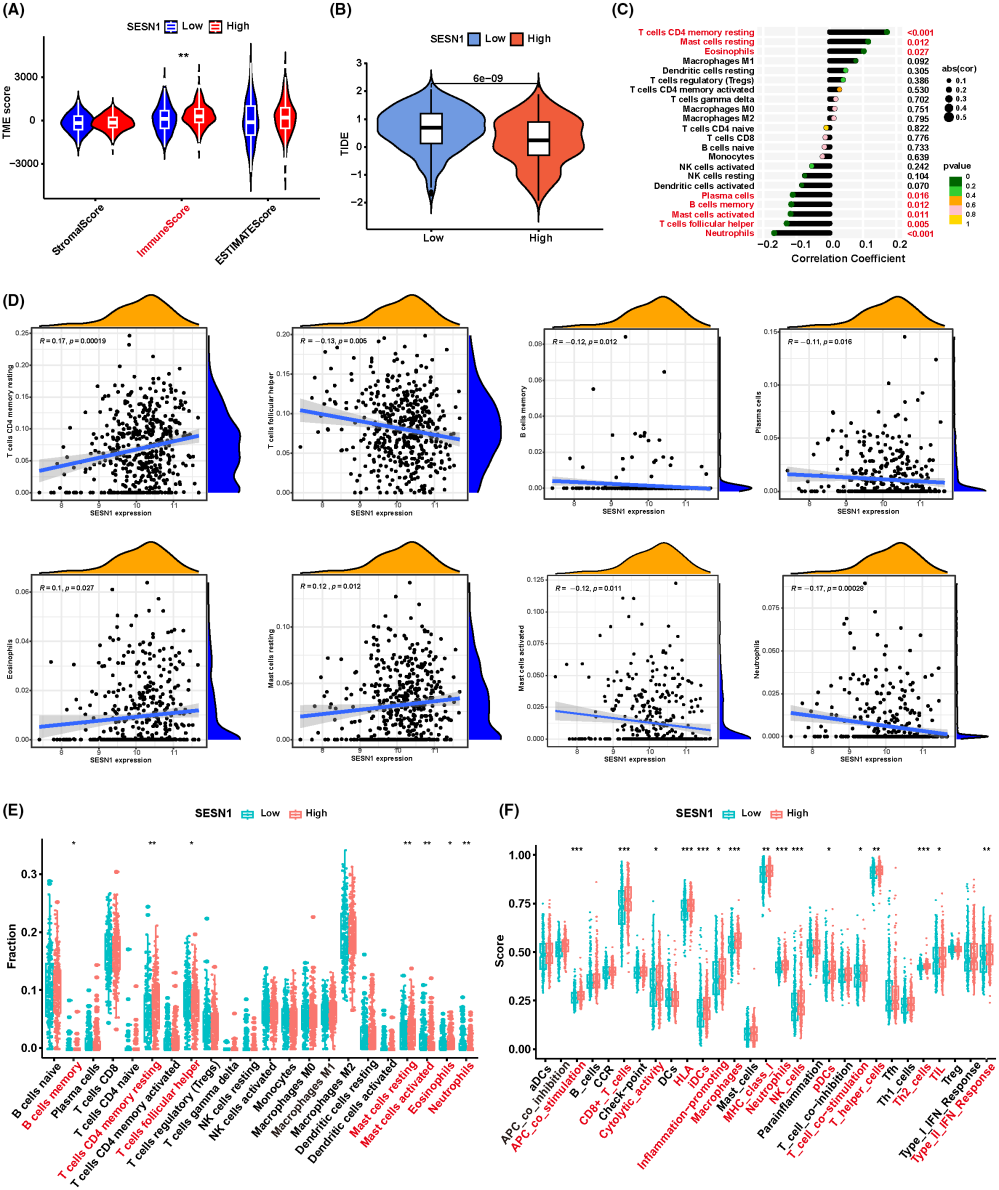
High expression of SESN1 indicated an active immune microenvironment. The stromal score, immune score, the ESTIMATE score (A) and the TIDE score (B) between high and low SESN1 expression. (C–E) The correlation of SESN1 with immune cells in the tumor microenvironment in NB. (F) The different immune function stages between SESN1 high and low expression groups. *p < 0.05, **p < 0.01, ***p < 0.001. SESN1 is a downstream target of MYCN and functions as a new tumor suppressor gene via Toll‐like receptor signaling pathway in neuroblastoma. High expression of SESN1 indicates a good prognosis and an active immune microenvironment for patients with NB.

KEGG enrichment analysis showed PD‐L1 expression and PD‐1 checkpoint pathway in cancer was another pathway enriched in all of the four cell lines transfected with SESN1 siRNA #1 and siRNA #2 (Figure 6B), and NB was a typical “cold tumor” with an impressive immune microenvironment. Immune microenvironment has been reported to regulate the metastasis of several tumors, including NB.^27^, ^28^, ^29^, ^30^, ^31^ To investigate the effect of SESN1 on the immune microenvironment, the tumor microenvironment (TME) scores were analyzed using “estimate” Packages in R software. High expression of SESN1 was associated with a higher stromal score, immune score, and ESTIMATE score (Figure 7A), especially for immune score (*p* < 0.01). Additionally, we performed TIDE (http://tide.dfci.harvard.edu/) for patients with NB with different expression levels of SESN1. Patients with low expression of SESN1 had significantly higher TIDE score (*p* < 0.001, Figure 7B), indicating an increase in immune escape potential and lower benefits from immune checkpoint suppressive therapy (ICI). Additionally, the expression of SESN1 was positively correlated with resting CD4+ T memory cells, resting mast cells, and eosinophils, but negatively correlated with plasma cells, memory B cells, activated mast cells, T follicular helper cells (Tfh), and neutrophils (*p* < 0.05, Figure 7C,E). Furthermore, we analyzed the relationship between SESN1 expression and the immune cells in TME. SESN1 expression was positively correlated with resting CD4+ T memory cells (*R* = 0.17, *p* = 0.00019), eosinophils (*R* = 0.1, *p* = 0.027), and resting mast cells (*R* = 0.12, *p* = 0.012), but negatively correlated with T follicular helper cells (*R* = −0.13, *p* = 0.005), memory B cells (*R* = −0.12, *p* = 0.012), plasma cells (*R* = −0.11, *p* = 0.016), activated mast cells (*R* = −0.12, *p* = 0.011), and neutrophils (*R* = −0.17, *p* = 0.00028) (Figure 7D). Additionally, we investigated the relationship between SESN1 expression and immune function. Patients with low SESN1 expression showed lower APC co‐stimulation, CD8+ T cells, cytolytic activity, HLA, iDCs, inflammation‐promoting, macrophages, MHC class I, neutrophils, NK cells, pDCs, T cell co‐stimulation, T helper cells, Th2 cells, TIL, and Type II IFN response (Figure 7F). These results suggested that patients with high SESN1 expression may have active immune reaction.

## 4 DISCUSSION

In the present study, we examined the function of SESN1 on NB. MYCN regulated the expression of SESN1 in NB cells, and knockdown SESN1 increased NB cell proliferation, cell migration, and cell invasion in vitro, and promoted NB tumor growth and shortened tumor‐bearing mice survival time in vivo. Overexpression of SESN1 had an anti‐tumor effect that decreased NB cell proliferation, cell migration, and cell invasion in vitro, and low expression of SESN1 means poor prognosis for patients with NB. Further analysis showed that TLR signaling pathway mediated the function of SESN1 on NB. Moreover, high expression of SESN1 indicated an active immune microenvironment.

Heterogeneity is one of the most important characteristics of NB, and the 5‐year survival rate of high‐risk patients is still less than 50% by now.^1^, ^2^, ^3^ There are about 20–30% of high‐risk patients have MYCN amplification, and approximately half of these patients have metastasis.^1^, ^4^ Researchers are working on to search for drugs that target MYCN and to clarify the mechanism of NB metastasis. Although there are currently no drugs specifically targeting MYCN, some drugs can target the downstream or upstream. BET is a family of proteins that regulate the transcription of many genes including MYCN. Notably, inhibitors targeting BET can block the transcription of MYCN and exert anti‐tumor effects in NB.^32^, ^33^, ^34^ Recent findings indicate that Aurora A Kinase (AURKA) is essential for MYCN stability and protects MYCN from degradation by proteasomes. Therefore, small molecular inhibitors targeting AURKA can indirectly inhibit the function of MYCN in NB.^35^, ^36^, ^37^ In the present study, we analyzed a microarray dataset of MYCN‐knockout NB cells (GSE121529)^22^ to identify new downstream targets of MYCN and verified that knockdown the expression of MYCN in MYCN‐amplified NGP cells increased SESN1 expression and overexpress MYCN in non‐MYCN‐amplified AS cells decreased SESN1 expression. This indicated that SESN1 may be a downstream target of MYCN.

SESN1 belongs to the Sestrin family, and SESN1 can be regulated by P53, FoxO3A, and FoxO1.^9^, ^12^, ^14^ Sestrin family members can be activated by various stresses, including oxidative stress or DNA damage, and play an important role in defense against reactive oxygen species (ROS), and can be regulated by mTOR and effect on cell apoptosis and autophagy.^38^, ^39^, ^40^ The other two members of Sestrin family, SESN2 and SESN3 have been reported to be involved in anoikis resistance in endometrial cancer cell lines.^15^ In the present study, knockdown SESN1 promoted cell proliferation, cell migration and cell invasion, and overexpressing SESN1 had opposite effects in vitro. Additionally, knockdown SESN1 promoted tumor growth and shortened the survival time in xenograft mouse model. Although SESN1 is a potential downstream target of MYCN, there was no significant difference in SESN1 functions between MYCN‐amplified and non‐MYCN‐amplified NB cells. Similarly, Wang et al. found that SESN1, a p53‐associated gene, was correlated with favorable clinical outcomes in pediatric NB not only for patients with MYCN amplification but also for patients with MYCN nonamplification.^41^

RNA‐Seq was performed to explore the mechanism of SESN1 in NB. Bioinformatic analysis showed that TLR signaling may mediate the function of SESN1 in NB. TLR signaling pathway is divided into MyD88‐dependent and MyD88‐independent pathway and plays essential roles in immunity.^18^, ^19^ Additionally, TLR signaling pathway plays an important role in cancer cell migration and metastasis. Hu et al. found that Biglycan (BGN) interacted with TLR signaling pathway, and enhanced endothelial cell migration and proliferation, and tube formation in gastric cancer.^42^ Zhang et al. found that TLR3 activation downregulated CXCR4, inhibited nasopharyngeal carcinoma (NPC) cell migration, and reduced the capacity of NPC cells to form metastasis in draining lymph nodes in vivo.^43^ Earl et al. demonstrated that silencing TLR4 in the mouse colon cancer cell line MC38 reduced the growth of tumors metastatic to the liver.^44^ In the present study, TLRs inhibitor HCQ reversed the function of SESN1 siRNA in NB cell proliferation, cell migration, and cell invasion, which may be via a MyD88‐dependent pathway.

NB is a typical “cold tumor” with an impressive immune microenvironment, and immune microenvironment has been reported to regulate the metastasis of several tumors, including NB.^27^, ^28^, ^29^, ^30^, ^31^ Zhang et al. found that TME in the brain of patients with lung cancer with brain metastasis was characterized by reduced antigen presentation and B/T cell function, increased numbers of neutrophils and M2‐type macrophages, immature microglia, and reactive astrocytes.^45^ Shani et al. found that targeting IL33 inhibited lung metastasis in breast cancer by attenuating immune cell recruitment and type 2 immunity in vivo.^46^ Moreover, a study using patients' samples and in vivo mouse model showed that patients with NB had suppressed tumor immune microenvironment, which was associated with chemoresistance and metastasis.^47^, ^48^ Tan et al. found that immune checkpoint inhibitors (ICIs) efficacy in patients with advanced triple‐negative breast cancer (TNBC) may be predicted by 12 mutant ctDNA genes, including SESN1.^49^ Balázs et al. found that radiotherapy accentuated the immune suppression of head and neck cancer patients via changing regulatory T cells and CTLA4 and PD‐1 expression on CD4 cells in the peripheral blood, and expression of the FXDR, SESN1, GADD45, DDB2, and MDM2 were altered in the peripheral blood cells of patients after radiotherapy.^50^ In the present study, KEGG enrichment analysis of the RNA‐seq data showed that PD‐L1 expression and PD‐1 checkpoint pathway in cancer was enriched in the four NB cell lines transfected with SESN1 siRNA #1 and siRNA #2. Further analysis of the role of SESN1 in the immune microenvironment of patients with NB showed that patients with high SESN1 expression may have an active immune reaction. How SESN1 affects the TIME of NB needs to be further study and we will focus on that in the future study.

In this study, we investigated the roles and mechanism of SESN1 in NB. SESN1 functions as a new tumor suppressor gene in NB via a MyD88‐dependent TLR signaling pathway. Overall, the findings of this study suggest that SESN1 and TLR signaling pathway may be potential new therapeutic targets for NB. Additionally, high expression of SESN1 indicates an active immune microenvironment, suggesting that SESN1 could be a potential immunotherapeutic target.

## FUNDING INFORMATION

This work was supported by the National Natural Science Foundation of China (No. 82002641, 82272940), the “Xingliao Talents Program” of Liaoning Province (XLYC2008010), Science and Technology Program of Liaoning Province (2022JH2/20200075), the 345 Talent Project and Shengjing Scholar Project of Shengjing Hospital.

## CONFLICT OF INTEREST STATEMENT

The authors declare no conflict of interest.

## Supporting information


Figures S1–S5

## Data Availability

The data of CRISPR‐Cas9 system knockout MYCN expression in NB cells were obtained from gene expression omnibus (GEO) (http://www.ncbi.nlm.nih.gov/geo/) at GSE121529. Clinical datasets (NBL, E‐MTAB‐8284, and GSE40710) were downloaded from TARGET (https://www.cancer.gov/ccg/research/genome‐sequencing/target), ArrayExpress (https://www.ebi.ac.uk/biostudies/arrayexpress), and GEO (https://www.ncbi.nlm.nih.gov/geo/) databases. The other datasets used and/or analyzed during the current study are available from the corresponding author upon reasonable request.
