# Supplementary material for: SESN1 functions as a new tumor suppressor gene via Toll‐like receptor signaling pathway in neuroblastoma
Source: CNS Neurosci Ther. 2024 Mar 22;30(3):e14664. doi: 10.1111/cns.14664 (PMC10958400; doi:10.1111/cns.14664)
Supplement: Supplementary file 1 — Figures S1–S5 [file CNS-30-e14664-s001.zip › cns14664-sup-0001-Supplementary information.docx]

**Supplementary information**

**Supplementary figure legends**

**Supplementary figure 1 A.** The top ten differentially expressed genes were shown ranking by degree score based on PPI analysis. **B.** GO analysis and KEGG enrichment analysis of SESN1 was done. The relationship between the expression of SESN1 and eventfree survival probability or overall survival probability of patients with NB was analyzed via R2 database (**C**), NBL datasets, E-MTAB-8284 datasets, and GSE40710 datasets (**D).**

**Supplementary figure 2 A.** Xenograft mice models bearing tumors (AS, SY5Y, BE2, NGP) were build, the weight of mice was measured once a week, and the mean weight changes of each group were shown. Means, SD.

**Supplementary figure 3 A.** The expression of SESN1 in different groups: female and male, age<18mon and age ≥18mon, with or without MYCN amplification, low or high risk group, different INSS stages, favor histopathology (FH) or unfavor histopathology (UFH), tumor progression and non-progression, and death from disease or not.

**Supplementary figure 4 A.** NB cells (AS, SY5Y, BE2, NGP) were treated with different concentration of HCQ (0, 10, 15, 20, 25, 30µM), the cell confluence was calculated based on the phase-contrast images by using IncuCyte Zoom software. Means, SD.

**Supplementary figure 5** Trans-well migration assay (**A**) and invasion assay (**B**) was performed after SESN1 siRNA#2 or/and MyD88 siRNAs transfection in SY5Y, BE2, and NGP cells, respective images were shown (left panel), and statistical analysis was done (right panel), **P*<0.05, ***P*<0.01, ****P*<0.001, ctrl siRNA-transfected cells vs. SESN1 siRNA#2-transfected cells, SESN1 siRNA#2 + MyD88 siRNA#1 or #2-transfected cells vs. SESN1 siRNA#2-transfected cells. Trans-well migration assay (C) and invasion assay (D) was performed SESN1 siRNA#2 transfection or/and HCQ treatment in SY5Y, BE2, and NGP cells, respective images were shown, and statistical analysis was done, ***P*<0.01, ****P*<0.001, ctrl siRNA-transfected cells vs. SESN1 siRNA#2-transfected cells, SESN1 siRNA#2 + HCQ-treated cells vs. SESN1 siRNA#2-transfected cells.
