# Supplementary material for: SESN1 functions as a new tumor suppressor gene via Toll‐like receptor signaling pathway in neuroblastoma
Source: CNS Neurosci Ther. 2024 Mar 22;30(3):e14664. doi: 10.1111/cns.14664 (PMC10958400; doi:10.1111/cns.14664)

Full unedited blot for Figure 1B

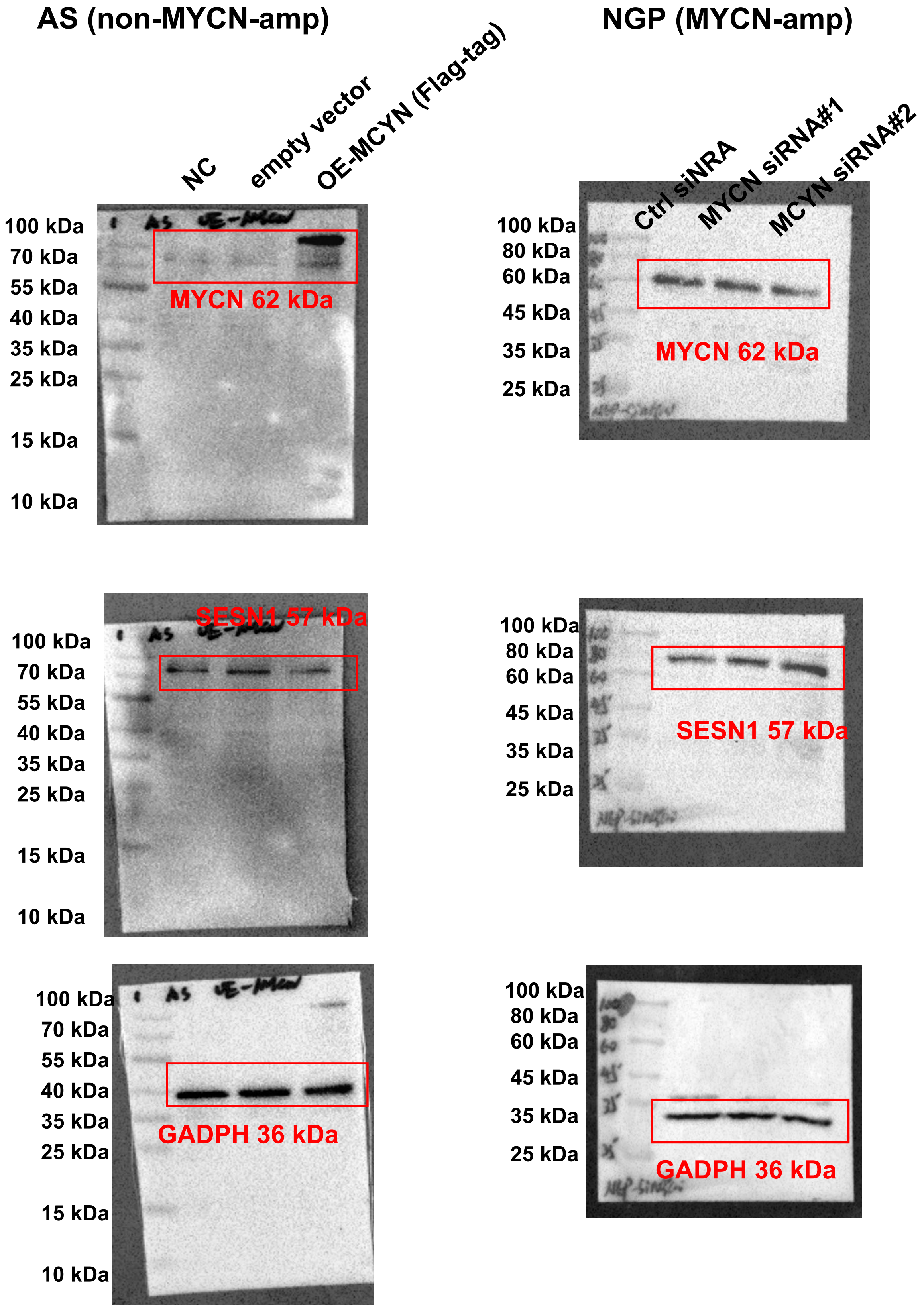

Full unedited blot for Figure 1C

AS

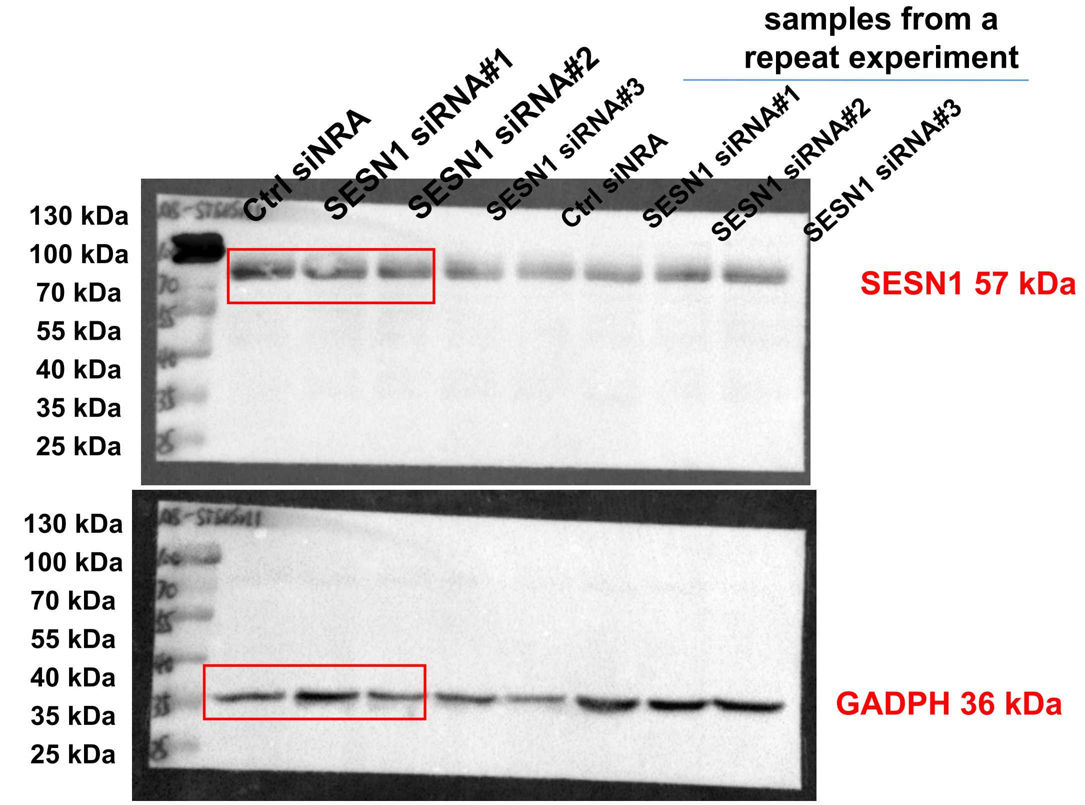

SY5Y

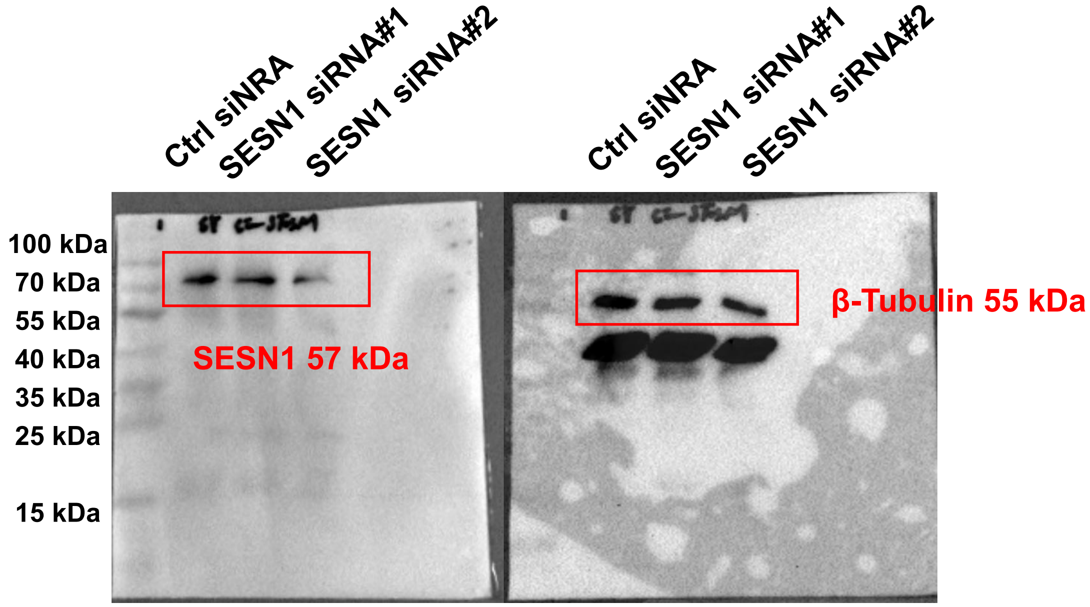

Full unedited blot for Figure 1C

BE2

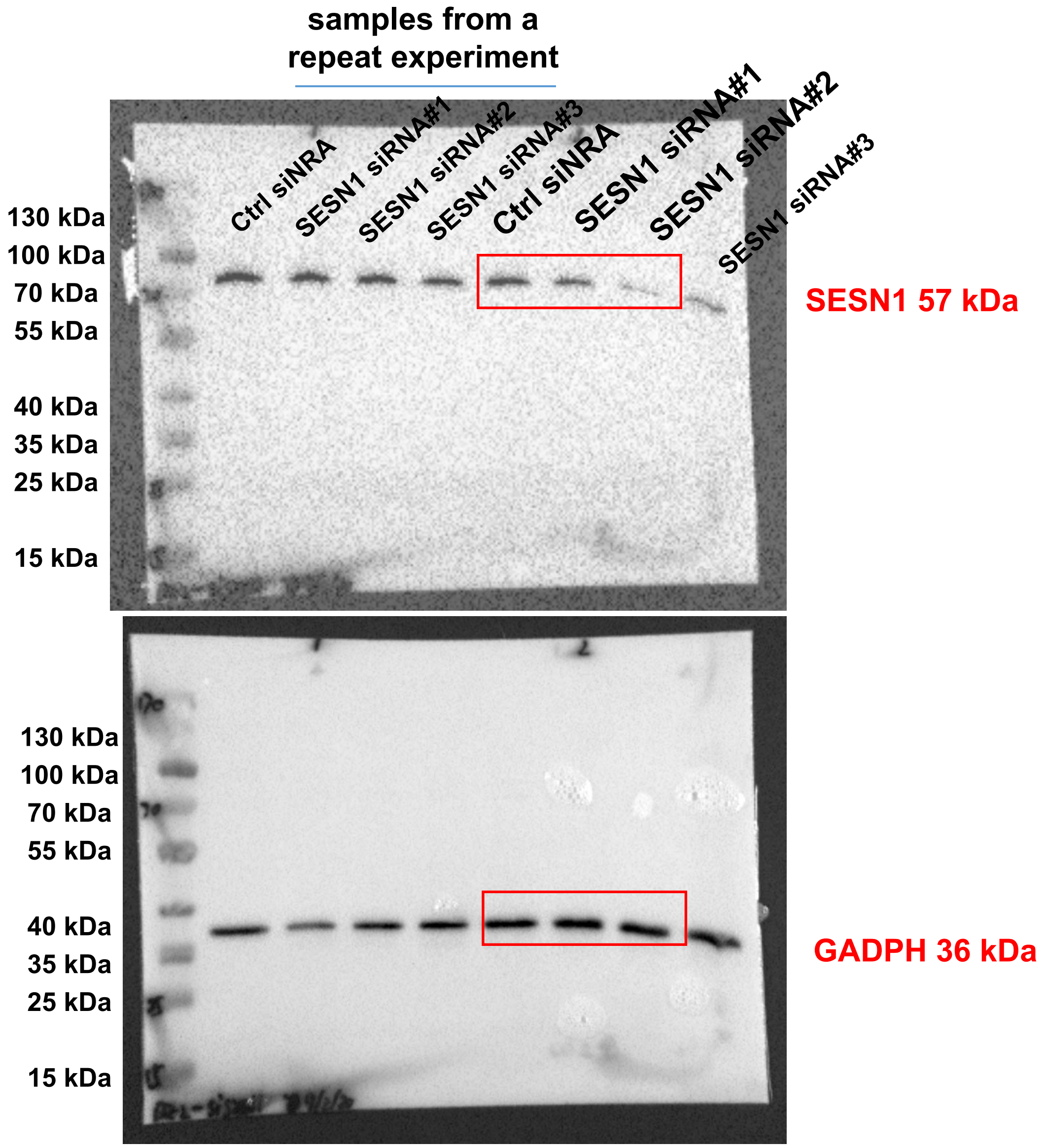

NGP

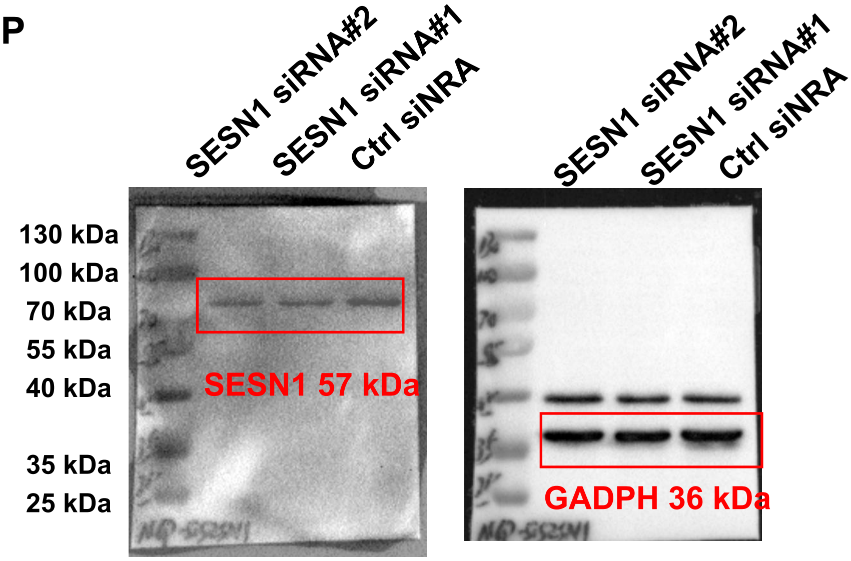

Full unedited blot for Figure 3A

AS

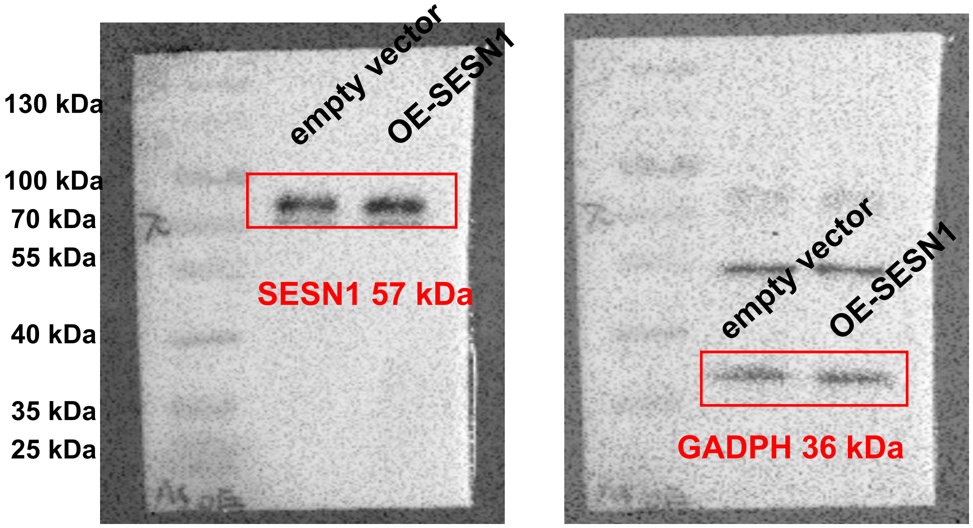

SY5Y

samples from a repeat experiment

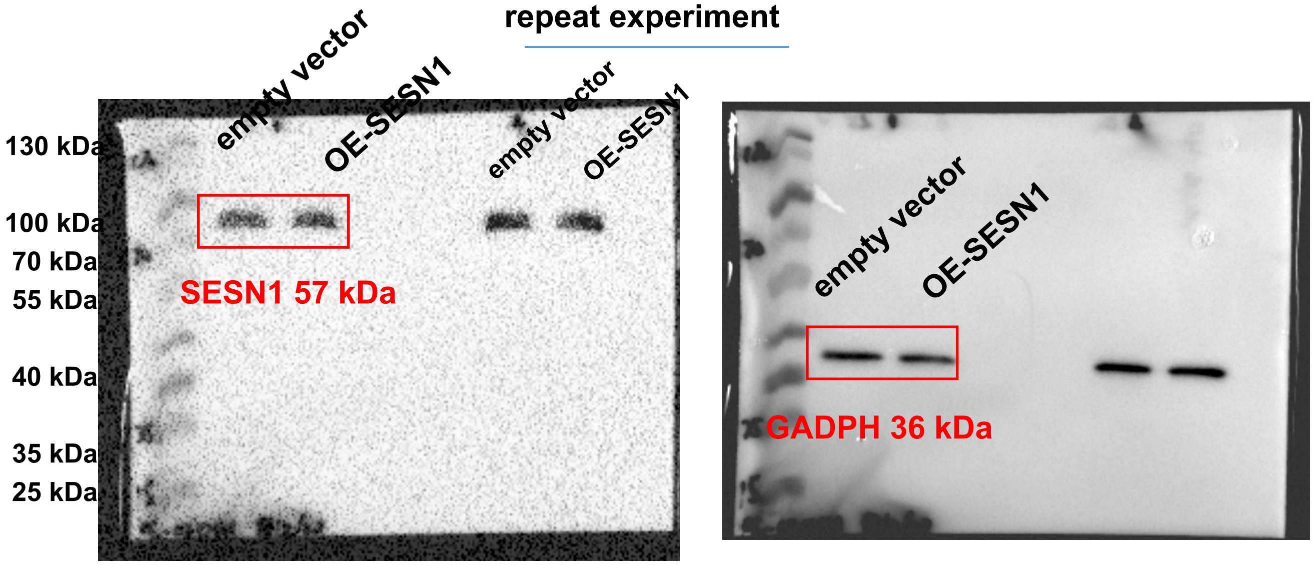

Full unedited blot for Figure 3A

BE2

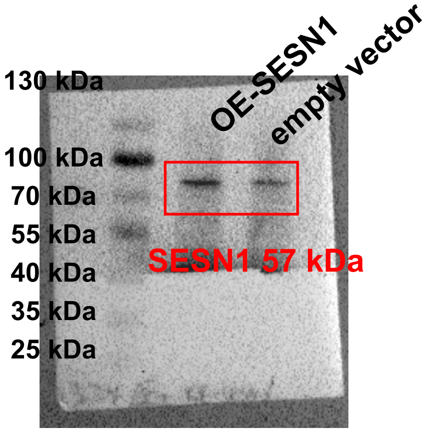

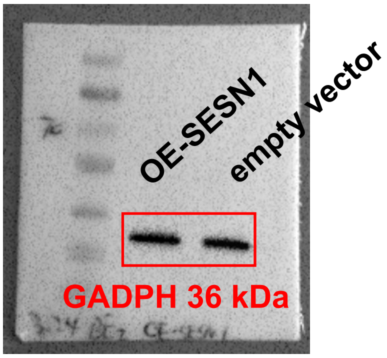

NGP

samples from a
repeat experiment

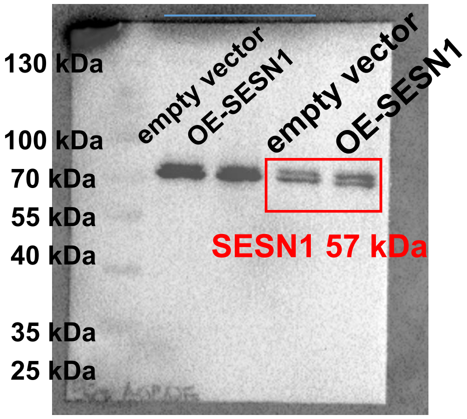

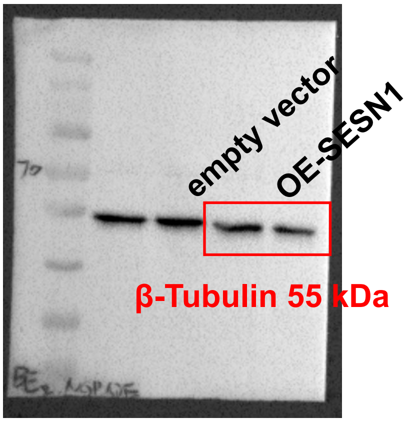

Full unedited blot for Figure 5D

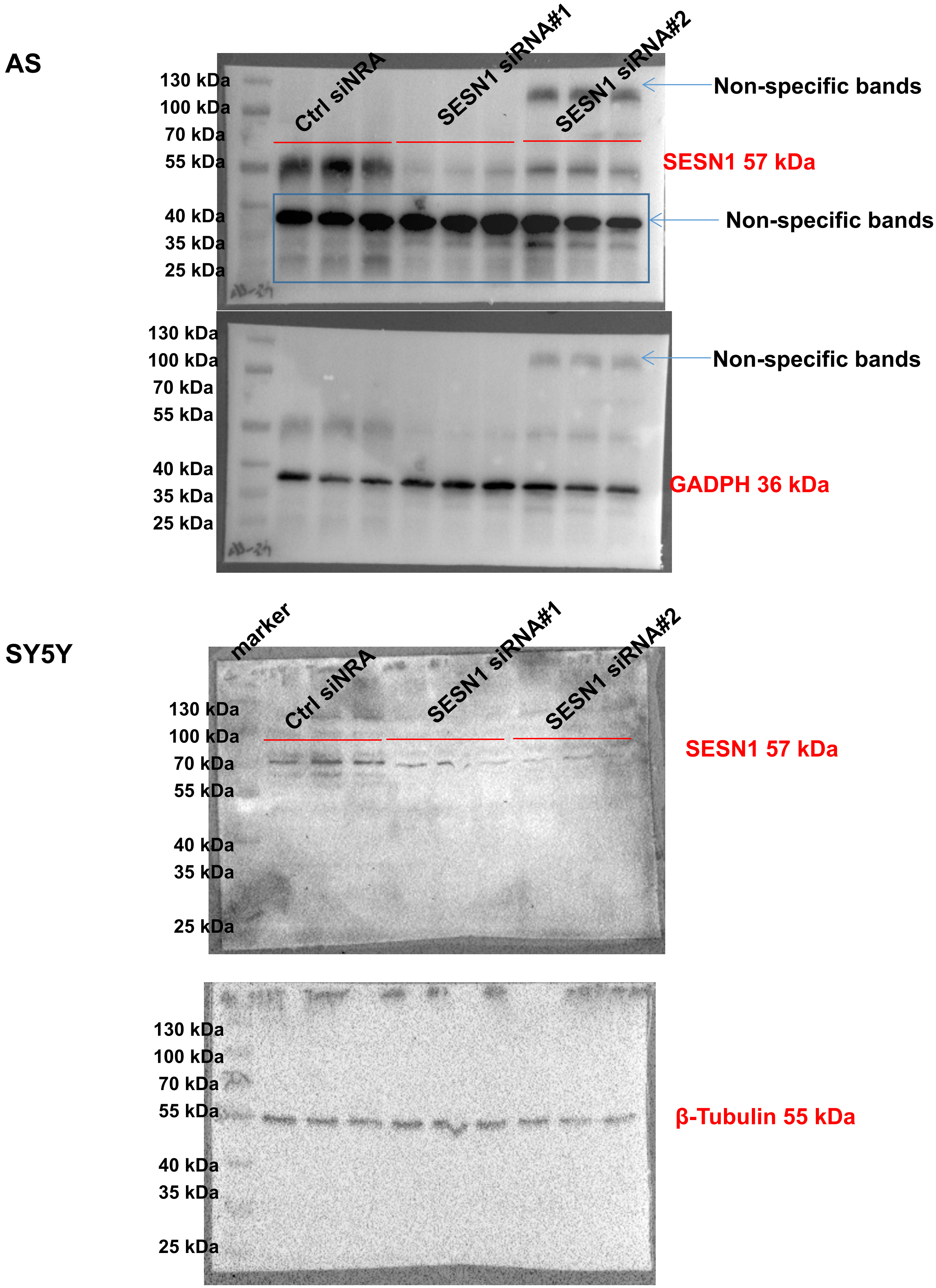

Full unedited blot for Figure 5D

BE2

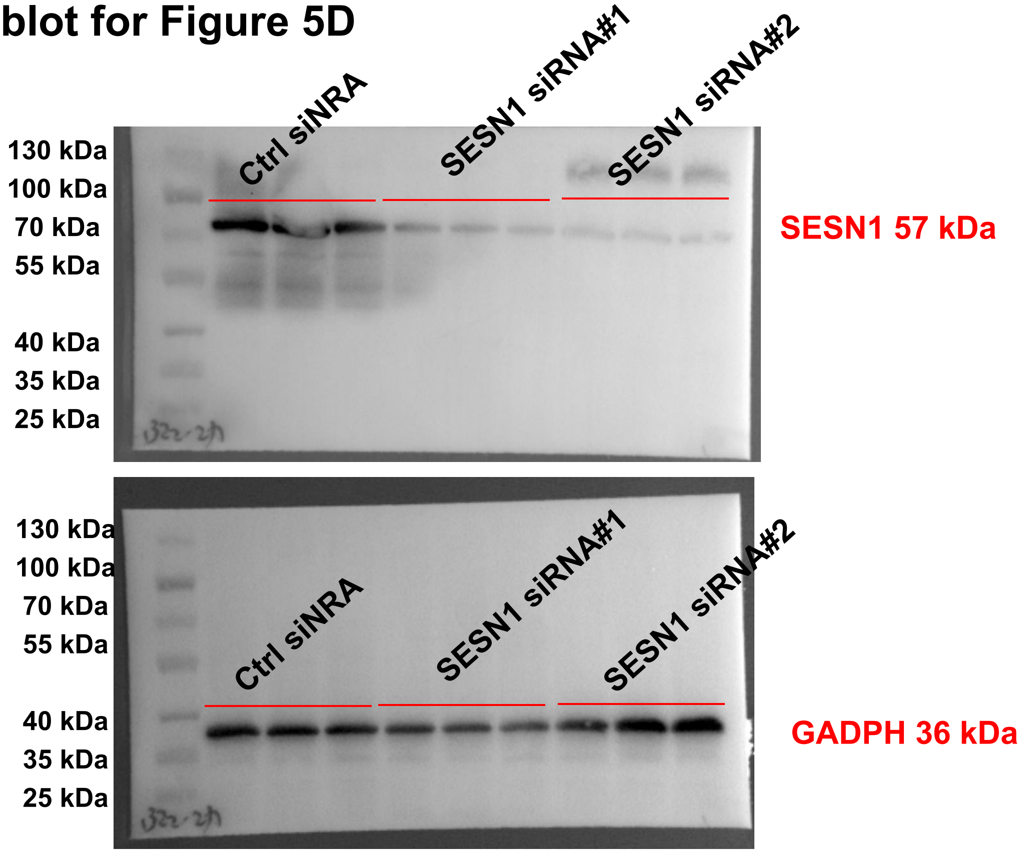

NGP

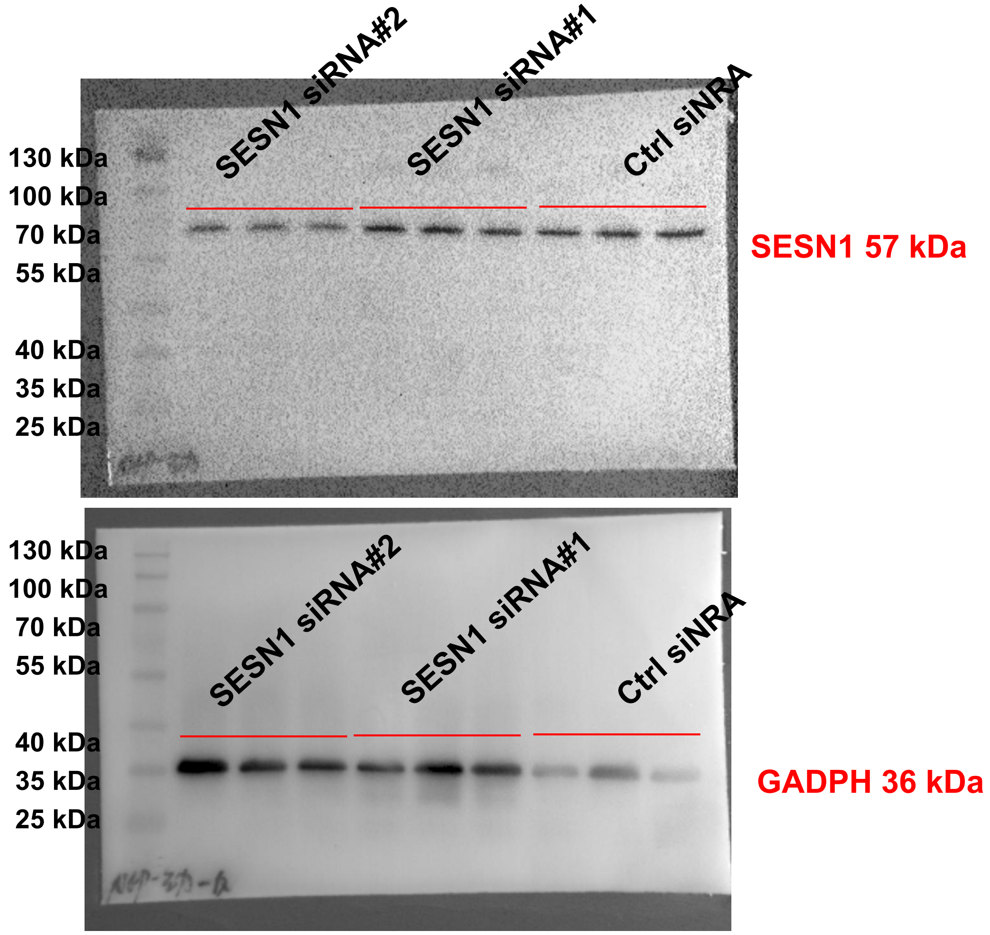

Full unedited blot for Figure 6C

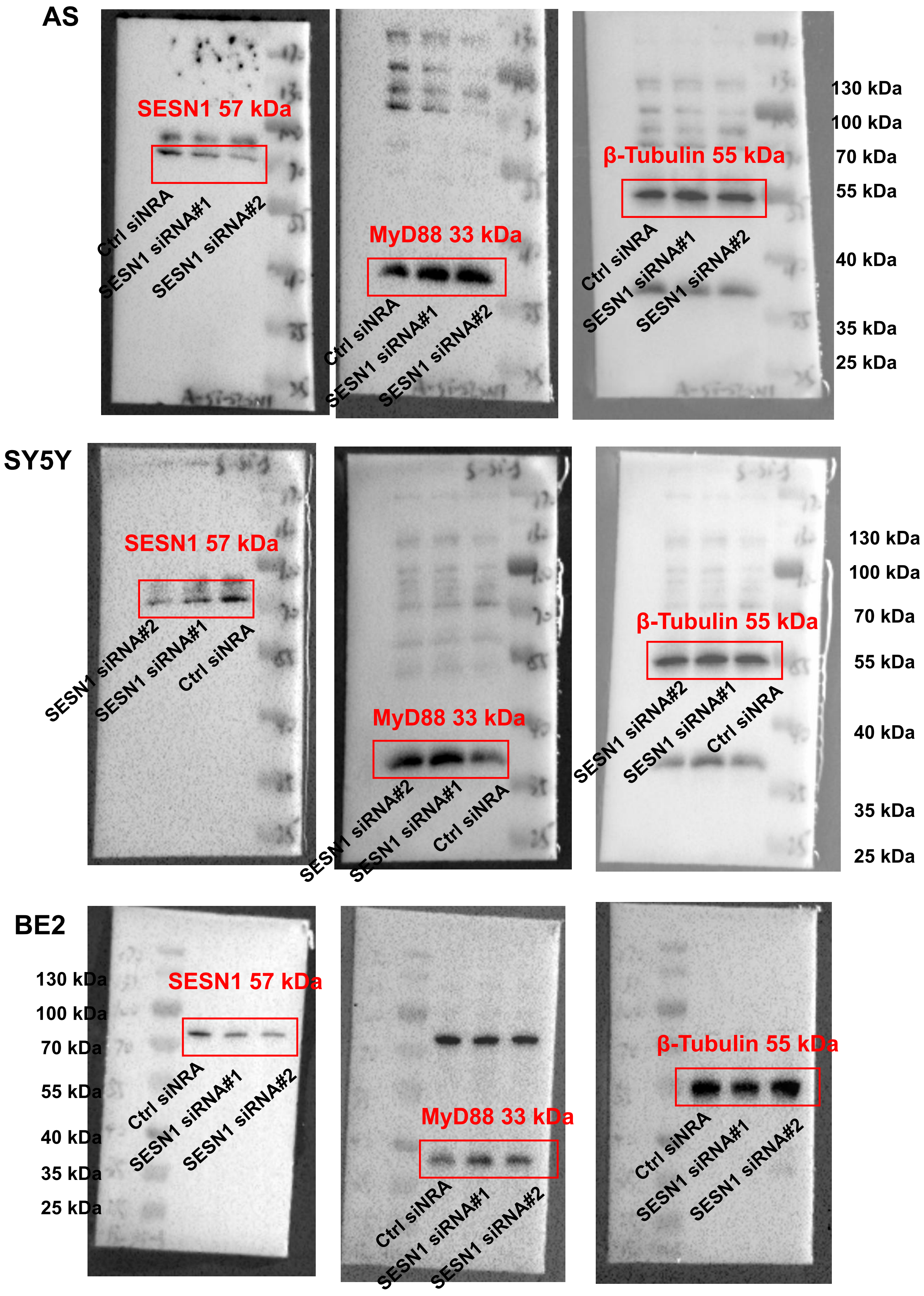

Full unedited blot for Figure 6D

AS

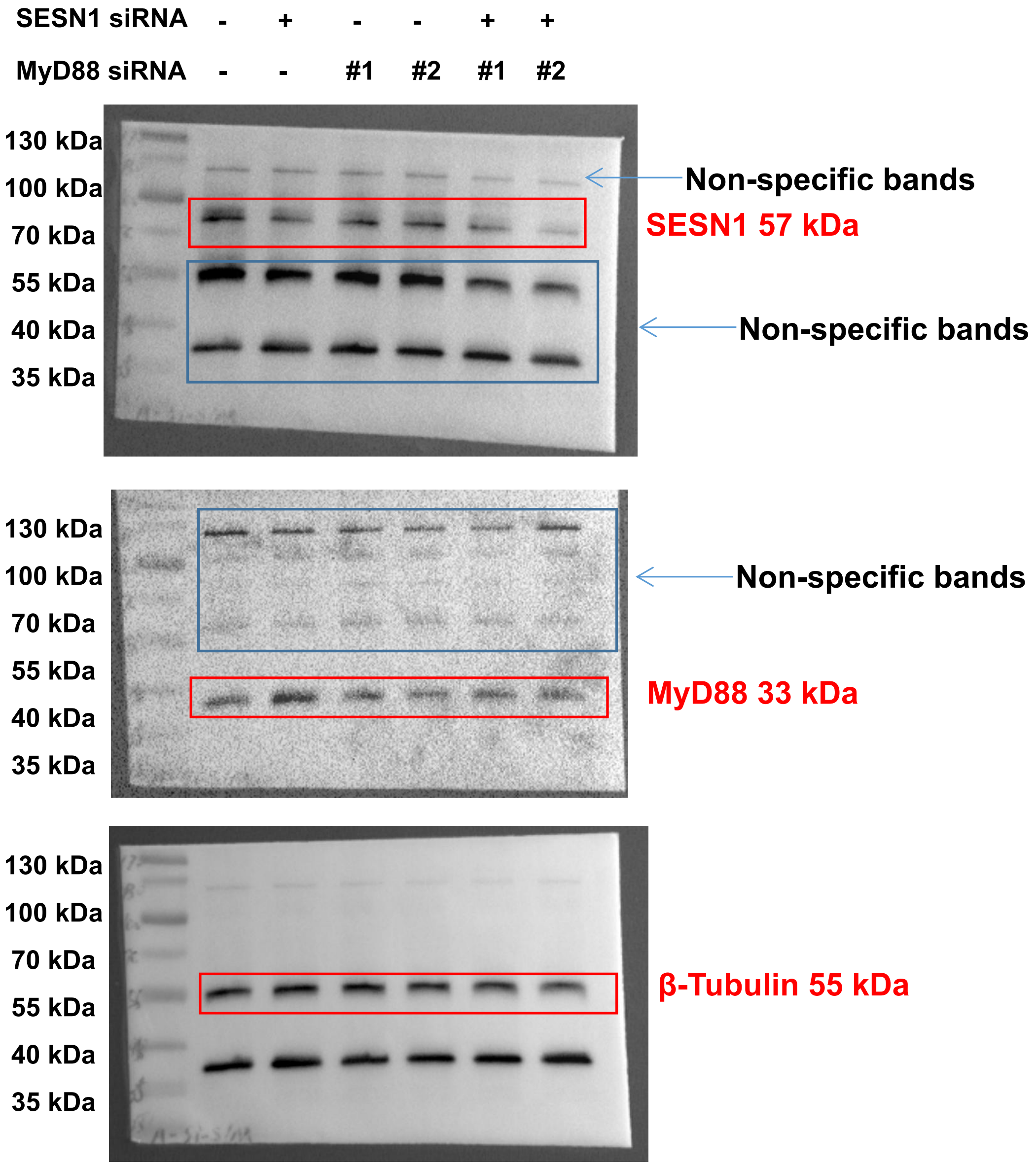

Full unedited blot for Figure 6E

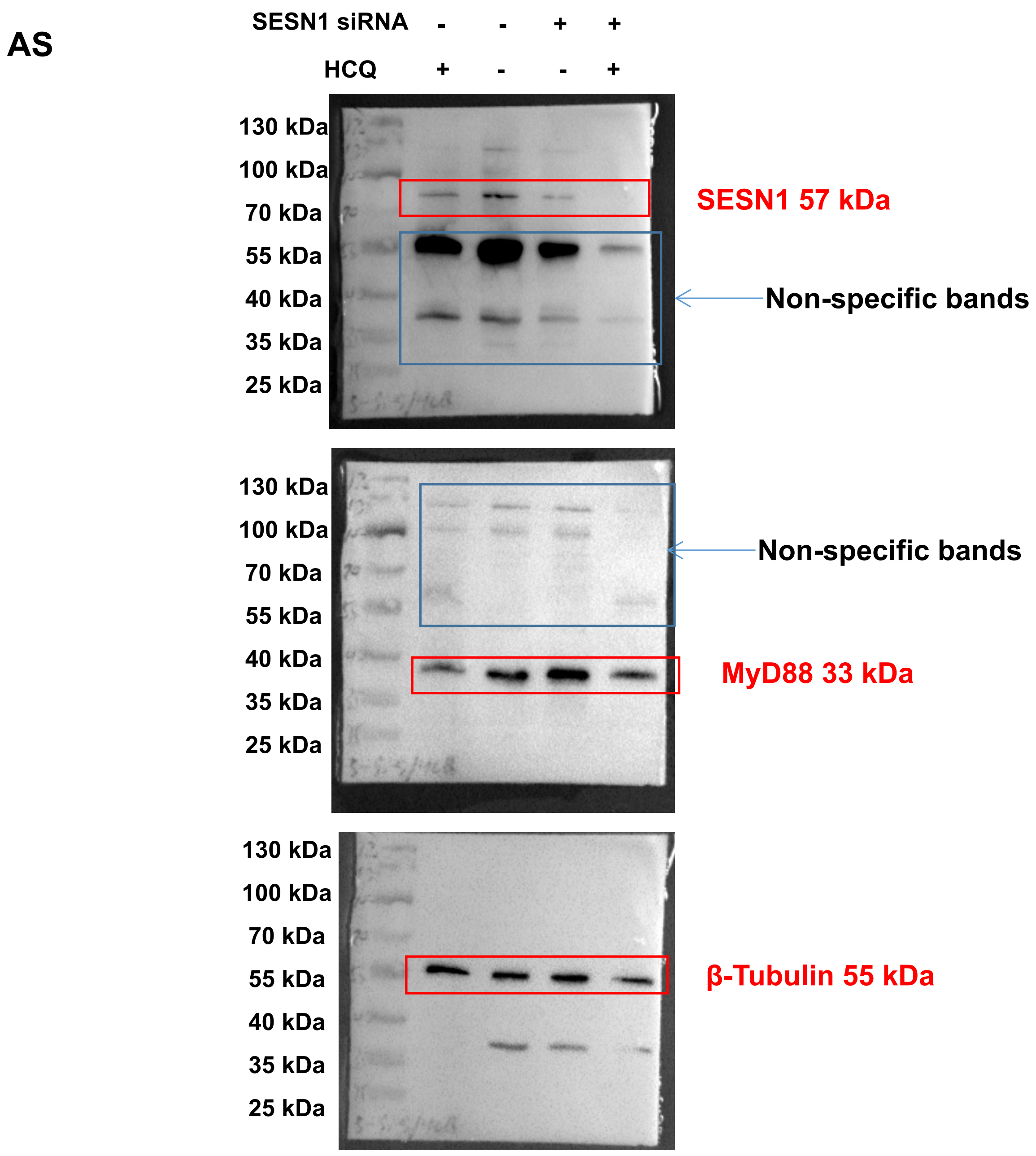

Supplement: Supplementary file 1 — Figures S1–S5 [file CNS-30-e14664-s001.zip › Original blot images.pdf]
