# Supplementary figures and images for: SESN1 functions as a new tumor suppressor gene via Toll‐like receptor signaling pathway in neuroblastoma
Source: CNS Neurosci Ther. 2024 Mar 22;30(3):e14664. doi: 10.1111/cns.14664 (PMC10958400; doi:10.1111/cns.14664)

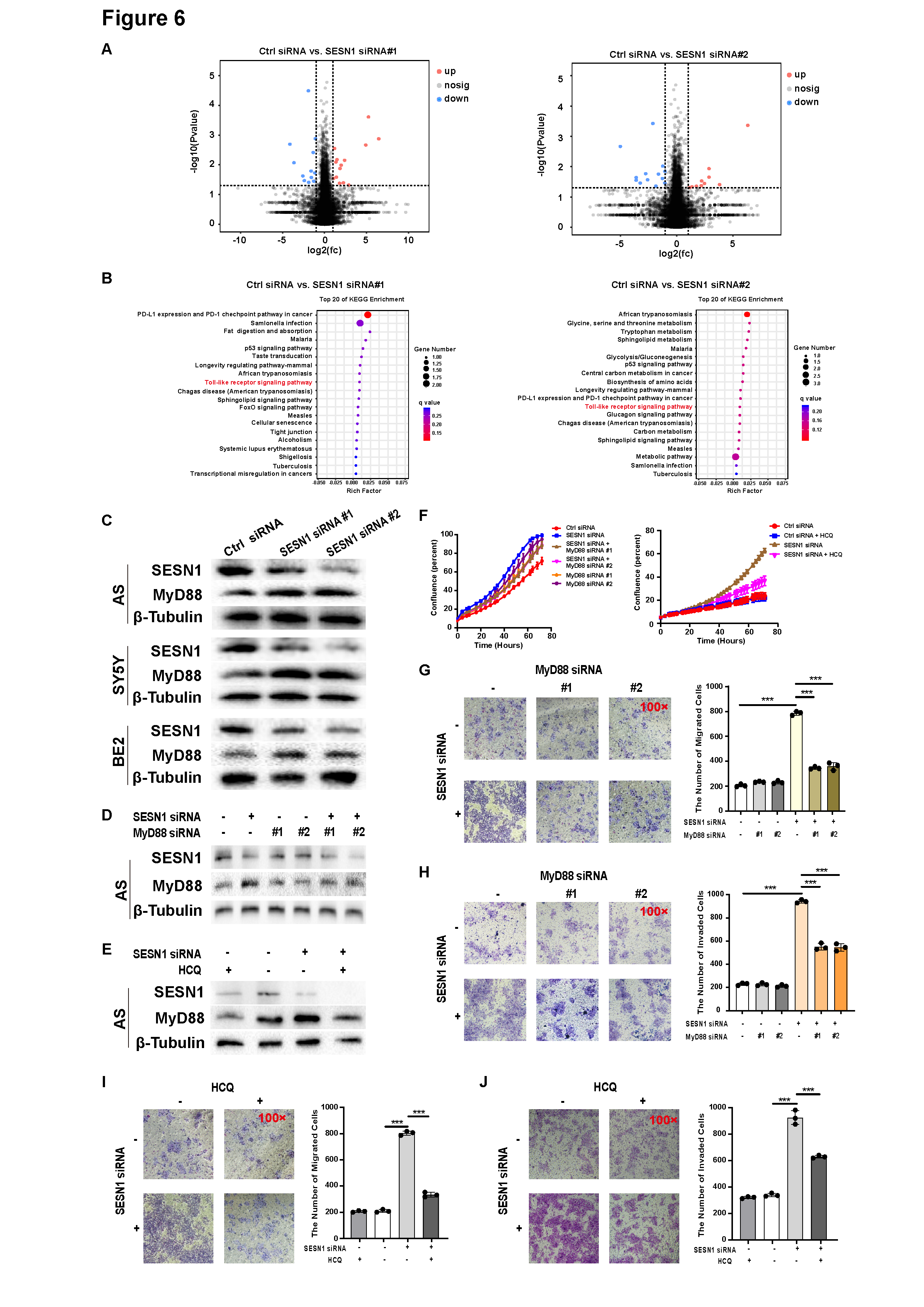

Supplement: Supplementary file 1 — Figures S1–S5 [file CNS-30-e14664-s001.zip › Re-revised Figure 6.tif]

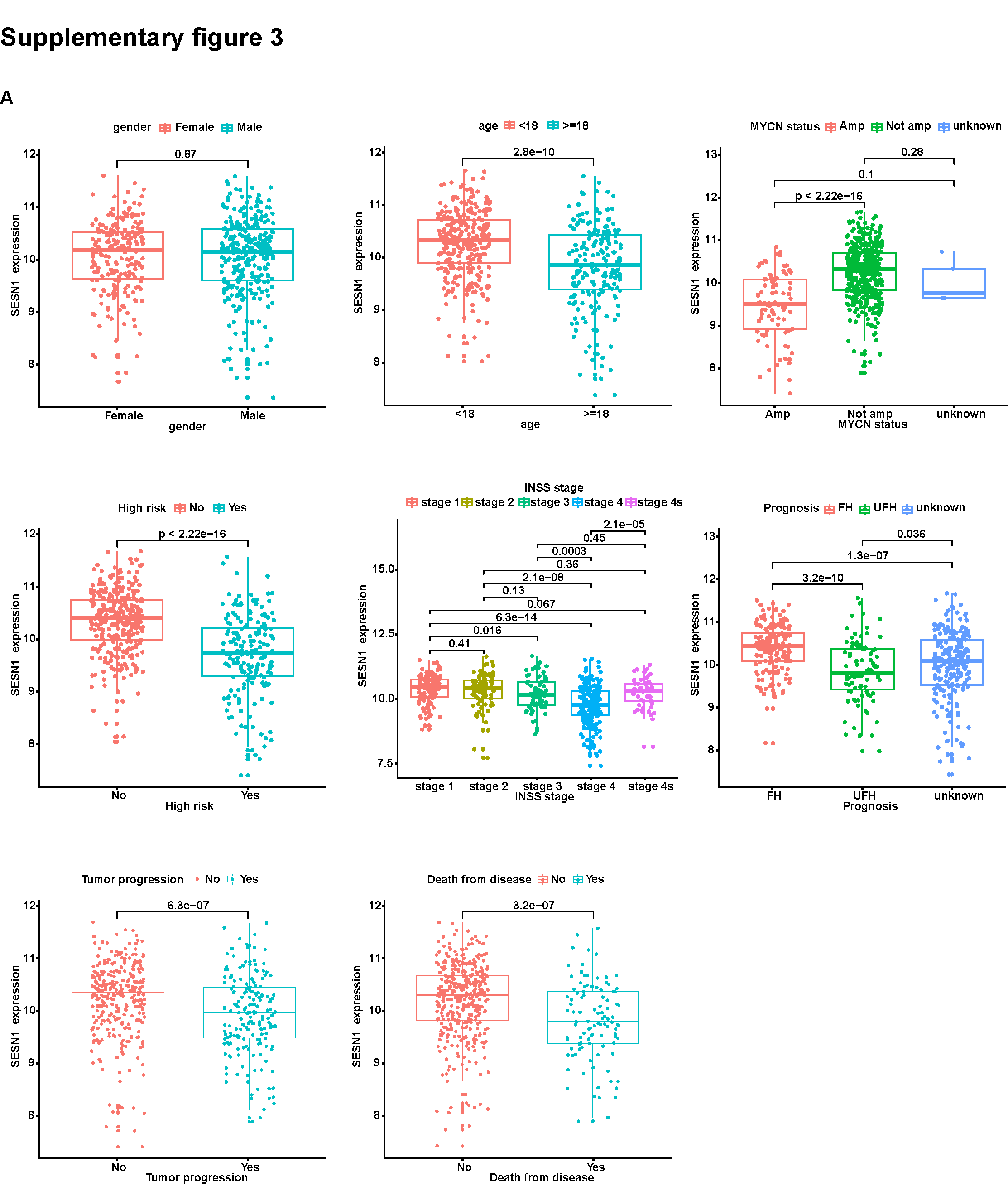

Supplement: Supplementary file 1 — Figures S1–S5 [file CNS-30-e14664-s001.zip › Revised Supplementary Figure 3.tif]

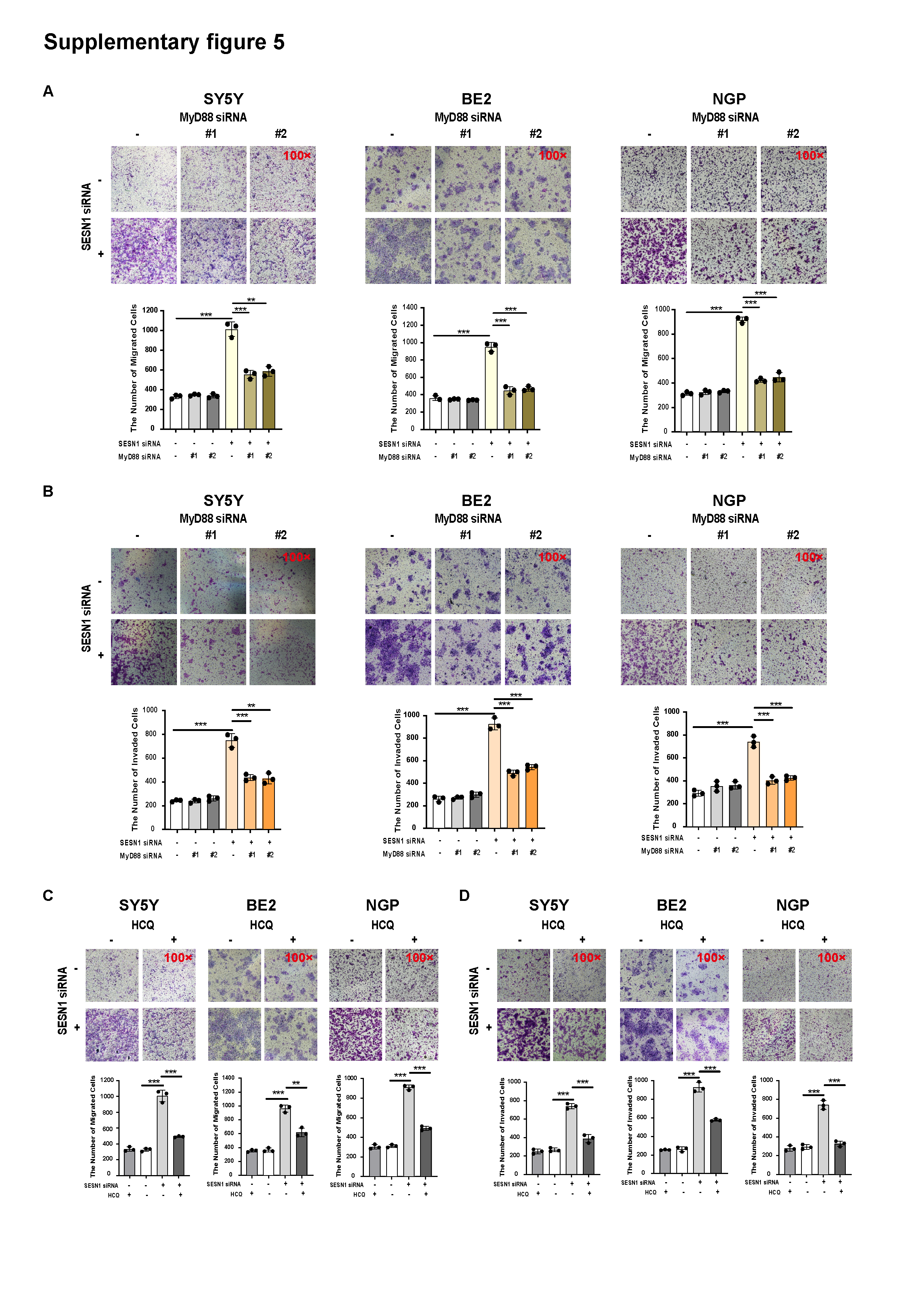

Supplement: Supplementary file 1 — Figures S1–S5 [file CNS-30-e14664-s001.zip › Revised supplementary Figure 5.tif]

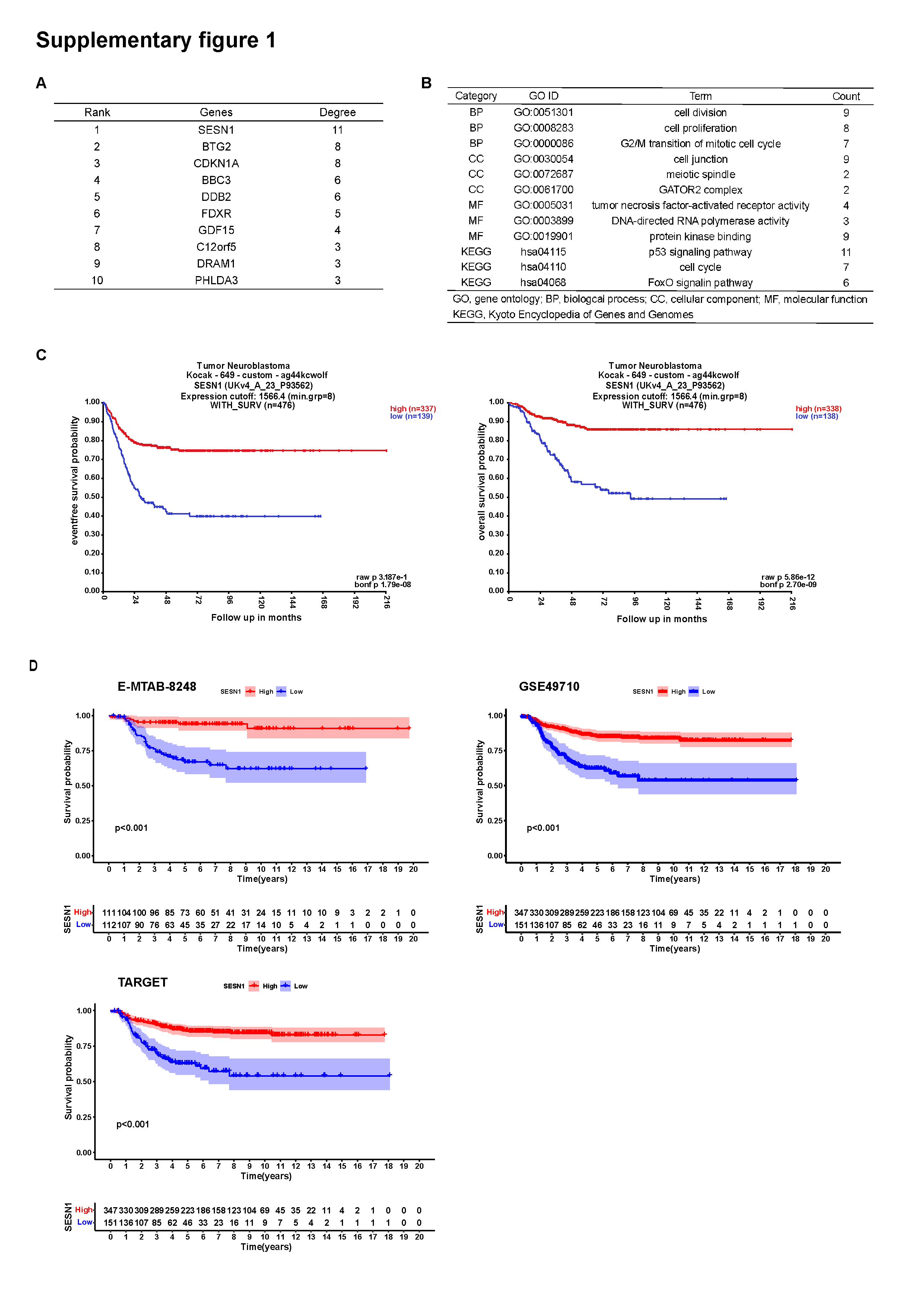

Supplement: Supplementary file 1 — Figures S1–S5 [file CNS-30-e14664-s001.zip › supplementary figure 1.tif]

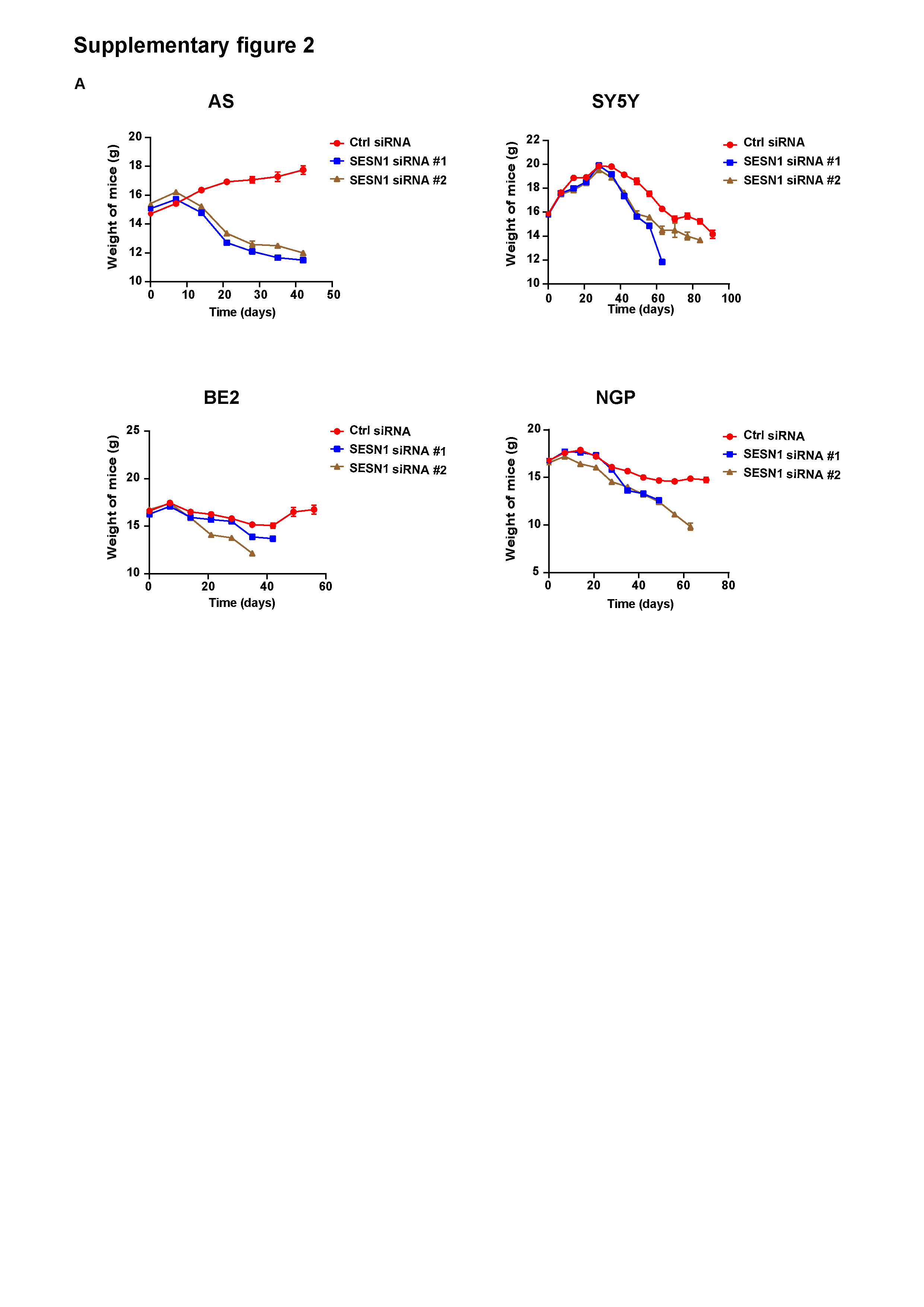

Supplement: Supplementary file 1 — Figures S1–S5 [file CNS-30-e14664-s001.zip › supplementary figure 2.tif]

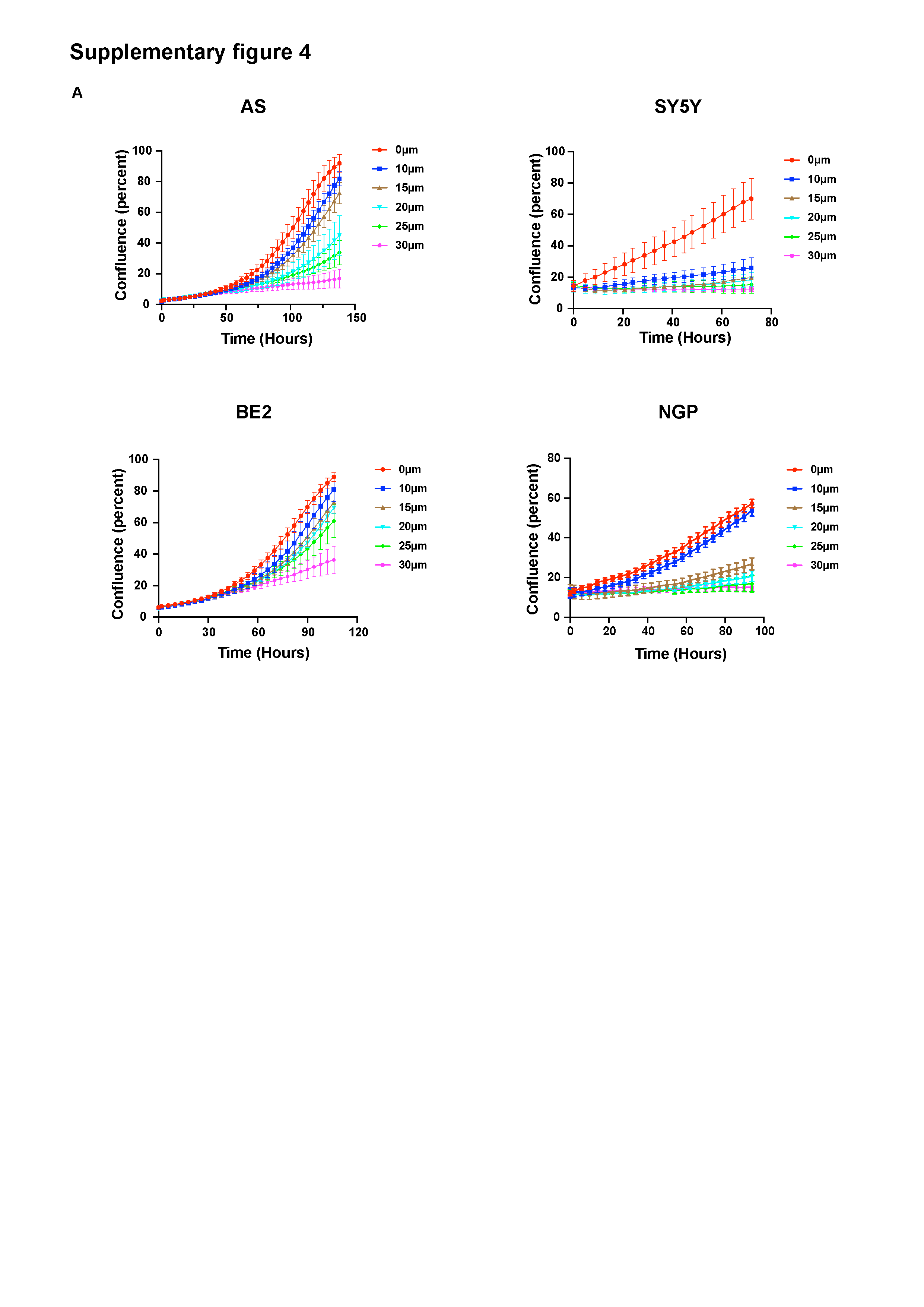

Supplement: Supplementary file 1 — Figures S1–S5 [file CNS-30-e14664-s001.zip › supplementary figure 4.tif]
